# *Brucella* Downregulates Tumor Necrosis Factor-α to Promote Intracellular Survival *via* Omp25 Regulation of Different MicroRNAs in Porcine and Murine Macrophages

**DOI:** 10.3389/fimmu.2017.02013

**Published:** 2018-01-17

**Authors:** Xiaomao Luo, Xiujuan Zhang, Xingchen Wu, Xuefeng Yang, Cong Han, Zhengyu Wang, Qian Du, Xiaomin Zhao, Shan-Lu Liu, Dewen Tong, Yong Huang

**Affiliations:** ^1^College of Veterinary Medicine, Northwest A&F University, Yangling, China; ^2^Center for Retrovirus Research, The Ohio State University, Columbus, OH, United States; ^3^Viruses and Emerging Pathogens Program, Infectious Diseases Institute, The Ohio State University, Columbus, OH, United States; ^4^Department of Veterinary Biosciences, The Ohio State University, Columbus, OH, United States; ^5^Department of Microbial Infection and Immunity, The Ohio State University, Columbus, OH, United States

**Keywords:** Omp25, tumor necrosis factor-α, miRNA, macrophage, *Brucella suis*

## Abstract

*Brucella* spp. impedes the production of pro-inflammatory cytokines by its outer membrane protein Omp25 in order to promote survival and immune evasion. However, how Omp25 regulates tumor necrosis factor (TNF-α) expression in different mammalian macrophages remains unclear. In this study, we investigated the potential mechanisms by which Omp25 regulates TNF-α expression and found that Omp25-deficient mutant of *B. suis* exhibited an enhanced TNF-α expression compared with wild-type (WT) *B. suis*, whereas ectopic expression of Omp25 suppressed LPS-induced TNF-α production at both protein and mRNA levels in porcine alveolar macrophages (PAMs) and murine macrophage RAW264.7 cells. We observed that Omp25 protein as well as WT *B. suis* upregulated miR-146a, -181a, -181b, and -301a-3p and downregulated TRAF6 and IRAK1 in both PAMs and RAW264.7 cells, but separately upregulates miR-130a-3p in PAMs and miR-351-5p in RAW264.7. The upregulation of miR-146a or miR-351-5p attenuated TNF-α transcription by targeting TRAF6 and IRAK1 at the 3′ untranslated region (UTR), resulting in inhibition of NF-kB pathway, while upregulation of miR-130a-3p, -181a, or -301a-3p correlated temporally with decreased TNF-α by targeting its 3′UTR in PAMs or RAW264.7 cells. In contrast, inhibition of miR-130a-3p, -146a, -181a, and -301a-3p attenuated the inhibitory effects of Omp25 on LPS-induced TNF-α in PAMs, while inhibition of miR-146a, -181a, -301a-3p, and -351-5p attenuated the inhibitory effects of Omp25 in RAW264.7, resulting in an increased TNF-α production and decreased intracellular bacteria in both cells. Taken together, our results demonstrate that *Brucella* downregulates TNF-α to promote intracellular survival *via* Omp25 regulation of different microRNAs in porcine and murine macrophages.

## Introduction

Brucellosis is one of the most widespread zoonotic diseases throughout the world, especially in developing countries (1). Monocytes and macrophages are the primary targets of *Brucella*, where *Brucella* replicate and cause persistent infection in human or livestock (2, 3). Meanwhile, the clearance of intracellular *Brucella* is dependent on the activated macrophages firstly *in vivo* (4). Thus, the activity of macrophages is very critical for the resistance of *Brucella* infection (5). As a crucial cytokine, tumor necrosis factor (TNF)-α can activate macrophages and protect human and animals from persistent *Brucella* infection (6, 7). However, the production of TNF-α is severely impaired in patients and domestic animals infected with *Brucella* species (8–10). Thus, *Brucella* can evade the innate immunity and establish long-lasting infection in dysfunctional macrophages, resulting in tissue lesions (11). Identifying the key component of *Brucella*, as well as understanding the underlying mechanisms that disturb the TNF-α expression in macrophages of different mammals is therefore essential.

Omp25 and Omp31 are main virulence-related factors of *Brucella* and show certain role in contribution to the intracellular survival of *Brucella* and chronic infection (12, 13). Compared to their parental wild-type (WT) strains, Omp25-deficient *Brucella melitensis, Brucella abortus, or Brucella ovis* mutants show an attenuated virulence in the infected murine model and ruminant hosts (14–16). WT *B. melitensis* or *Brucella suis* infection exhibits a compromised ability to induce TNF-α (6, 17), yet Omp25-deficient *B. suis* shows an enhanced ability to induce TNF-α in human macrophages. These data suggest Omp25 plays an important role in inhibiting TNF-α production, yet the molecular mechanism of how *Brucella* Omp25 inhibits TNF-α production are unclear and remain to be determined.

In this study, we examined the roles of Omp25 in regulation of *Brucella*- or LPS-induced TNF-α expression and investigated the underlying mechanisms in porcine alveolar macrophages (PAMs) and murine macrophages (RAW264.7 cells). We analyzed several miRNAs that regulate TNF-α expression at both transcriptional and posttranscriptional levels. We found that Omp25-induced miR-146a, miR-181a, and miR-301a-3p regulate TNF-α in both PAMs and mouse RAW264.7 cells, whereas Omp25-induced miR-130a-3p and miR-351-5p specifically regulate TNF-α expression in porcine and murine macrophages, respectively. Importantly, suppression of these miRNAs not only attenuated the inhibition of Omp25 on TNF-α expression but also promoted the clearance of intracellular bacteria as well as TNF-α production in *Brucella*-infected cells upon subsequent stimulation. This study provides new insights for understanding the mechanisms of macrophage dysfunction in different species during *Brucella* infection.

## Materials and Methods

### Cell Culture

Porcine alveolar macrophage cell line (PAM, CRL-2843), mouse macrophage RAW264.7 cell line (RAW264.7, TIB-71), and human embryonic kidney 293 T cell line (HEK-293T, CRL-3216) were all purchased from American Type Tissue Culture (ATCC). Mouse RAW264.7 cells and HEK-293T cells were cultured in Dulbecco’s modified Eagle’s medium (DMEM) supplemented with 10% heat-inactivated fetal bovine serum (FBS). PAMs were maintained in Roswell Park Memorial Institute (RPMI) 1640 medium (Invitrogen) with 10% heat-inactivated FBS, sodium pyruvate, nonessential amino acids. All cells were cultured in a fully humidified atmosphere containing 5% CO_2_ at 37°C. The cells before passage 15 were seeded into six-well plates (2–6 × 10^5^ cells per well) and cultured in medium for 12 h, and the cells were precultured to 60% confluency before infection or transfection.

### Bacterial Strains and Lentiviral Particle Preparation

Omp25- and Omp31-deficient mutants (Δ*omp25* and Δ*omp31*) were obtained using suicide pCVD442-derived plasmids (Addgene, Cambridge, MA, USA) carrying *omp25* and *omp31* gene interrupted by kanamycin (Kan; 50 µg/ml) resistance gene as previously described (18, 19). Trans complementation of the Δ*omp25 B. suis* with plasmid pBBR1MCS-2 included the native *omp25* gene (GenBank accession no. U39397.1) from *B. suis* recovered the expression of Omp25 (Δ*omp25*-*omp25 B. suis*). Δ*omp31 B. suis* complemented in trans with the native *omp31* gene (GenBank accession no. AF366063.1) from *B. suis* and cloned into pBBR1MCS-2 recovered the Omp31 expression (Δ*omp31*-*omp31 B. suis*). *B. suis* 1330 (23444, ATCC) and its mutants were cultured in 50 ml of tryptic soy broth (Difco Laboratories) medium with constant agitation at 37°C. The target gene *omp25* was amplified by PCR from *B. suis* 1330 using *omp25* specific primers with Flag-tagged (Table S1 in Supplementary Material). The full-length *omp25* was cloned into PCDH-CMV-MCS-EF1-copGFP (Addgene, Cambridge, MA, USA) vector according to the manufacturer’s instructions, which was then co-transfected with psPAX2 and PMD2-VSV (Addgene) plasmids into HEK-293T cells for 48 h to generate individual lentivirus particles. Supernatants containing lentiviral particles were harvested and concentrated, and viral titer was determined through infecting HEK-293T cells (20). In this study, all procedures involving live *Brucella* experiments were performed in a biosafety level 3 facility in accordance with the approved protocols.

### *Brucella* Infection and Intracellular Survival Assays

A standard gentamicin protection assay was carried out to determine the number of intracellular *B. suis* bacteria. PAMs and mouse RAW264.7 cells were seeded in six-well plates (5 × 10^5^ cells per well) and infected with *B. suis* 1330 or mutated *B. suis* strains at 50:1 multiplicity of infection (MOI) for 1 h. After incubation, cells were washed three times with phosphate-buffered saline (PBS) and further cultured with RPMI 1640 or DMEM with 10% heat-inactivated FBS containing 50 µg/ml gentamicin (Sigma) and 50 µg/ml of streptomycin (Sigma) to clear remaining extracellular bacteria. The infection of *Brucella* was performed as previously described (21).

For accounting the number of bacteria survived in PAMs and mouse RAW264.7 cells, infected cells were treated with 0.1 ml of 0.1% Triton X-100 (Sigma) in PBS for 5 min at 37°C, and lysates were diluted in PBS and plated onto tryptic soy agar to determine the colony-forming units (22).

### Enzyme-Linked Immunosorbent Assay (ELISA)

Porcine alveolar macrophages and mouse RAW264.7 cells were adhered to six-well plates followed by 100 MOIs of lentivirus or 50 MOIs of *B. suis* infection. Supernatants were harvested to measure TNF-α, IL-12 p40, IL-6, and IL-1β secretion by enzyme-linked immunosorbent assay (ELISA) kit (R&D) according to manufacturer’s instructions (23).

### Western Blotting

Cells were suspended in Radio Immunoprecipitation Assay lysis buffer (Thermo Scientific, PA, USA) supplemented with protease inhibitor (Sigma Aldrich). Cytosol and nuclear fractions were isolated according to manufacturer’s instruction (Thermo). Equivalent proteins were subjected to SDS-PAGE and transferred to polyvinyl difluoride membranes (Millipore Corp., Atlanta, GA, USA) for western blotting. After blocking membrane with 5% non-fat dry milk for 2 h, we incubated it with primary antibodies at 4°C overnight. Primary antibodies included anti-FLAG M2 (Sigma), anti-Omp25, anti-Omp31 and anti-β-actin (Wuhan boster Biotech), anti-phospho-IκB, anti-IκB, anti-p65, anti-TRAF6, anti-IRAK1, anti-IRAK2, and anti-Histone H3 (Cell Signaling Technology). HRP-conjugated secondary antibodies were incubated at room temperature for 1 h. ECL (Bio-Rad) was used for enhanced chemiluminescence detection according to the manufacturer’s instructions.

### Quantitative PCR

MicroRNAs and mRNA were quantified by quantitative polymerase chain reaction (Q-PCR), with total cellular RNA being isolated by TRIZOL (24). Briefly, RNA concentration and purity were measured using a NanoDrop spectrophotometer (Thermo). Reverse transcription of mRNA was performed using M-MLV reverse transcriptase with 2 µg of total RNA initially treated with DNase I, random primer, 1× first-strand buffer, RNase inhibitor, and DTT. Reverse transcription of microRNA was performed using 0.5 µg of total RNA initially treated with RNase inhibitor, stem–loop RT primers, oligo DT, 1× first-strand buffer, RNase inhibitor, M-MLV, and DTT (Invitrogen, Carlsbad, CA, USA). The specific primers of porcine TNF-α mRNA (GenBank accession no. NM_214022), porcine IL-12 p40 mRNA (GenBank accession no. NM_002187), porcine IL-6 mRNA (GenBank accession no. NM_214055.1), porcine IL-1β mRNA (GenBank accession no. NM_214399.1), mouse TNF-α mRNA (GenBank accession no. NM_013693), mouse IL-12 p40 mRNA (GenBank accession no. NM_001303244.1), mouse IL-6 mRNA (GenBank accession no. NM_031168.2), and mouse IL-1β mRNA (GenBank accession no. NM_008361.4) were showed in the Table S2 in Supplementary Material, and all of miRNAs GenBank accession numbers and primers were showed in the Table S3 in Supplementary Material. The relative levels of miRNAs and mRNA were analyzed by SYBR-green based Q-PCR using a Bio-Rad IQ5 Real-Time PCR System. The relative quantification of miRNA and mRNA were done using the ΔΔCt method (25).

### Bioinformatics for miRNA

miRNA targets were predicted using TargetScan (26). TNF-α of porcine and murine sequences were from the National Center for Biotechnology Information database, miRNAs predicted to bind to 3′ untranslated region (UTR) regions of porcine TNF-α, and murine TNF-α genes were selected by target analysis through the prediction algorithms TargetScan mouse and miRBase.

### Transfection of Mimics and Inhibitors

Cells were seeded overnight before transfection, allowed to reach 50% confluency by the time of transfection, and transfected with 100 nM miRNAs mimics, mimics control, miRNAs inhibitors (Anti-miRNA), miRNA inhibitors control (Table S4 in Supplementary Material) (GenePharma Biotechnology, Shanghai, China), or the mix of indicated miRNAs mimics, or the mix of indicated miRNAs inhibitors using lipofectamine 3000 (Invitrogen, USA), respectively. At 6 h post-transfection, the cells were refreshed in medium and infected with 100 MOIs of LV-Blank or lentivirus expressing Omp25 (LV-Omp25), or 50 MOIs of WT *B. suis* or Δ*omp25 B. suis* for indicated times in each experiment.

### Luciferase Reporter Assay

Porcine TNF-α promoter and murine TNF-α promoter sequence was amplified and cloned into report vector pGL3 plasmid (Promega Corporation, Madison, WI, USA) according to Harmon assay (27) using specific primers (Table S2 in Supplementary Material). PAMs and mouse RAW264.7 cells were transfected with a mixture of pGL-TNF-α or NF-κB activity reporter plasmid and pRL-TK renilla luciferase plasmid using lipofectamine 3000 (Invitrogen). PCI-neo, pCI-Omp31 or pCI-Omp25 was transfected at same time. Luciferase activities were measured 24 h later using the Dual-Luciferase^®^ reporter assay (Promega) according to the instructions of manufacturer.

The full-length 3′UTR of the porcine TNF-α gene or murine TNF-α gene were amplified from PAMs or RAW264.7 cells cDNA using specific primers (Table S2 in Supplementary Material) and cloned into pMIR-REPORT Luciferase vector (Ambion). The TNF-α 3′UTR containing the mutated ssc-miR-130a-3p target sequence (TTGCACT to AACGTGA), ssc-miR-181a target sequence (TGAATGT to ACTTACA), ssc-miR-181b target sequence (TGAATGT to ACTTACA), ssc-miR-301a-3p target sequence (TTGCACT to AACGTGA), mmu-miR-130a-3p target sequence (TTGCACT to AACGTGA), mmu-miR-181a target sequence (TGAATGT to ACTTACA), mmu-miR-181b target sequence (TGAATGT to ACTTACA), mmu-miR-301a-3p target sequence (TTGCACT to AACGTGA), and mmu-miR-351-5p target sequence (CTCAGGG to GAGTCCC) were cloned into the pMIR-REPORT Luciferase vector. The primers for mutants are also shown in Table S2 in Supplementary Material, HEK-293 cells were transfected with homologous wild or mutated type TNF-α 3′UTR firefly luciferase reporter plasmids and pTK-Renilla luciferase plasmids, together with mimics control, miR-130a-3p mimics, miR-181a mimics, miR-181b mimics, miR-301a-3p mimics or miR-351-5p mimics for 48 h, and Luciferase activity was measured.

### Statistical Analysis

The data were presented as mean ± SEM (SD). SPSS software was used to statistical analysis. Data of multiple groups were analyzed using one-way ANOVA followed by Bonferroni *post hoc* test, while comparisons between two groups were performed by unpaired Student’s *t*-test. Statistical significance was defined as *P* < 0.05.

## Results

### Omp25 Deficiency Attenuates the Inhibitory Effects of *B. suis* on TNF-α Production in PAMs and Mouse RAW264.7 Cells

**Figure 1:**
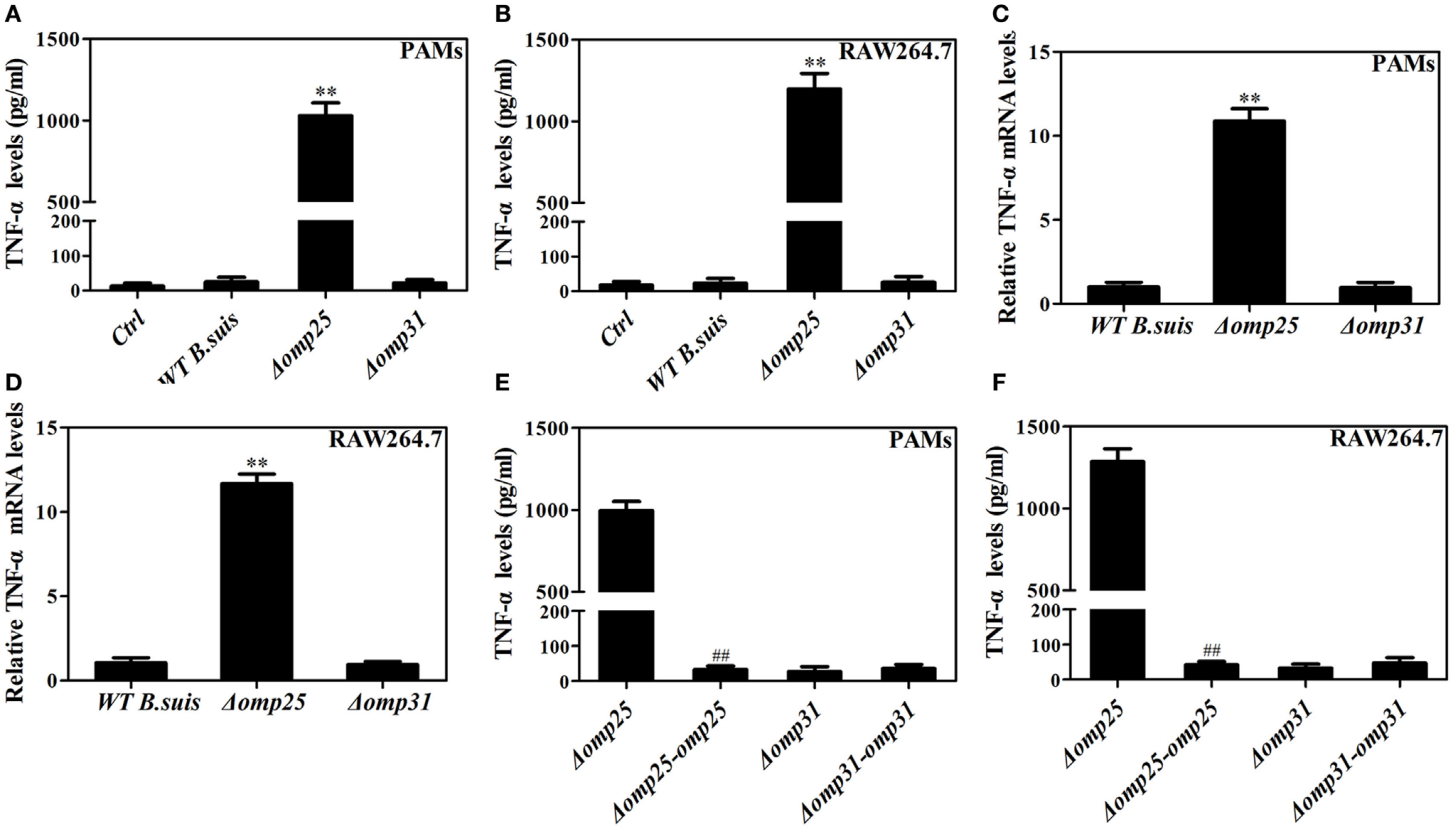
Deficiency of *omp25* enhances *B. suis*-induced tumor necrosis factor (TNF)-α production in porcine alveolar macrophages (PAMs) and mouse RAW264.7 cells. **(A,B)** PAMs and RAW264.7 cells were infected with wild-type (WT) *B. suis*, Omp25-deficient mutant (Δ*omp25 B. suis*), Omp31-deficient mutant (Δ*omp31 B. suis*) or were uninfected (ctrl), and TNF-α secretion was measured at 24 h post-infection in culture supernatants by enzyme-linked immunosorbent assay (ELISA). **(C,D)** PAMs and RAW264.7 cells were infected with WT *B. suis*, Δ*omp25*, or Δ*omp31* and cultured for 6 h, Q-PCR was used to measure TNF-α mRNAs levels. **(E,F)** PAMs and mouse RAW264.7 cells were infected with Δ*omp25*, the complemented Δ*omp25* strain of *B. suis* (Δ*omp25*-*omp25 B. suis*), Δ*omp31*, or the complemented Δ*omp31* strain of *B. suis* (Δ*omp31*-*omp31 B. suis*), followed by ELISA detection of TNF-α in culture supernatants. The results are mean ± SEM of three independent experiments. **P < 0.01 versus WT *B. suis***-**infected cells. ^##^*P* < 0.01 versus Δ*omp25 B. suis*-infected cells.

To determine the roles of Omp25 and Omp31 proteins in the intracellular survival and regulating TNF-α production in *Brucella* infection of macrophages, we analyzed the intracellular survival of Omp25-deficient mutant (Δ*omp25 B. suis*) and Omp31-deficient mutant (Δ*omp31 B. suis*), and compared the levels of TNF-α in WT *B. suis*, Δ*omp25 B. suis*, and Δ*omp31 B. suis-*infected PAMs or RAW264.7 cells. We found that although Omp25 and Omp31 deficiency did not affect the intracellular survival of *B. suis* within 24 h of infection, Δ*omp25-B. suis* showed a lower level of survival compared to WT *B. suis* and Δ*omp31 B. suis* at 48 h post-infection (Figure S1 in Supplementary Material). Of note, in both PAMs and mouse RAW264.7 cells, Δ*omp25 B. suis* infection induced more TNF-α production than did the WT *B. suis*, yet Δ*omp31 B. suis* infection did not show any difference from WT *B. suis* in TNF-α production (Figures 1A,B). Similarly, the level of TNF-α mRNA was higher in the Δ*omp25 B. suis*-infected cells compared to that of WT *B. suis* or Δ*omp31 B. suis*-infected cells (Figures 1C,D). Omp25-deficient mutant (Δ*omp25 B. suis*) and Omp31-deficient mutant (Δ*omp31 B. suis*) were then transduced with Omp25 and Omp31 expression plasmid, respectively, in order to complement the Omp25 and Omp31 expression (Figure S2 in Supplementary Material). Results showed that the TNF-α production of Δ*omp25*-complemented-*B. suis*-infected cells was significantly lower than that of Δ*omp25 B. suis*-infected cells, while Δ*omp31*-complemented-*B*. *suis*-infected cells was not different from Δ*omp31 B. suis*-infected cells in TNF-α production (Figures 1E,F). These results demonstrate that the presence of Omp25, rather than Omp31, inhibits *B. suis*’s ability to induce TNF-α in the PAMs and mouse RAW264.7 cells.

### Omp25 Inhibits LPS-Induced TNF-α Production in PAMs and Mouse RAW264.7 Cells

**Figure 2:**
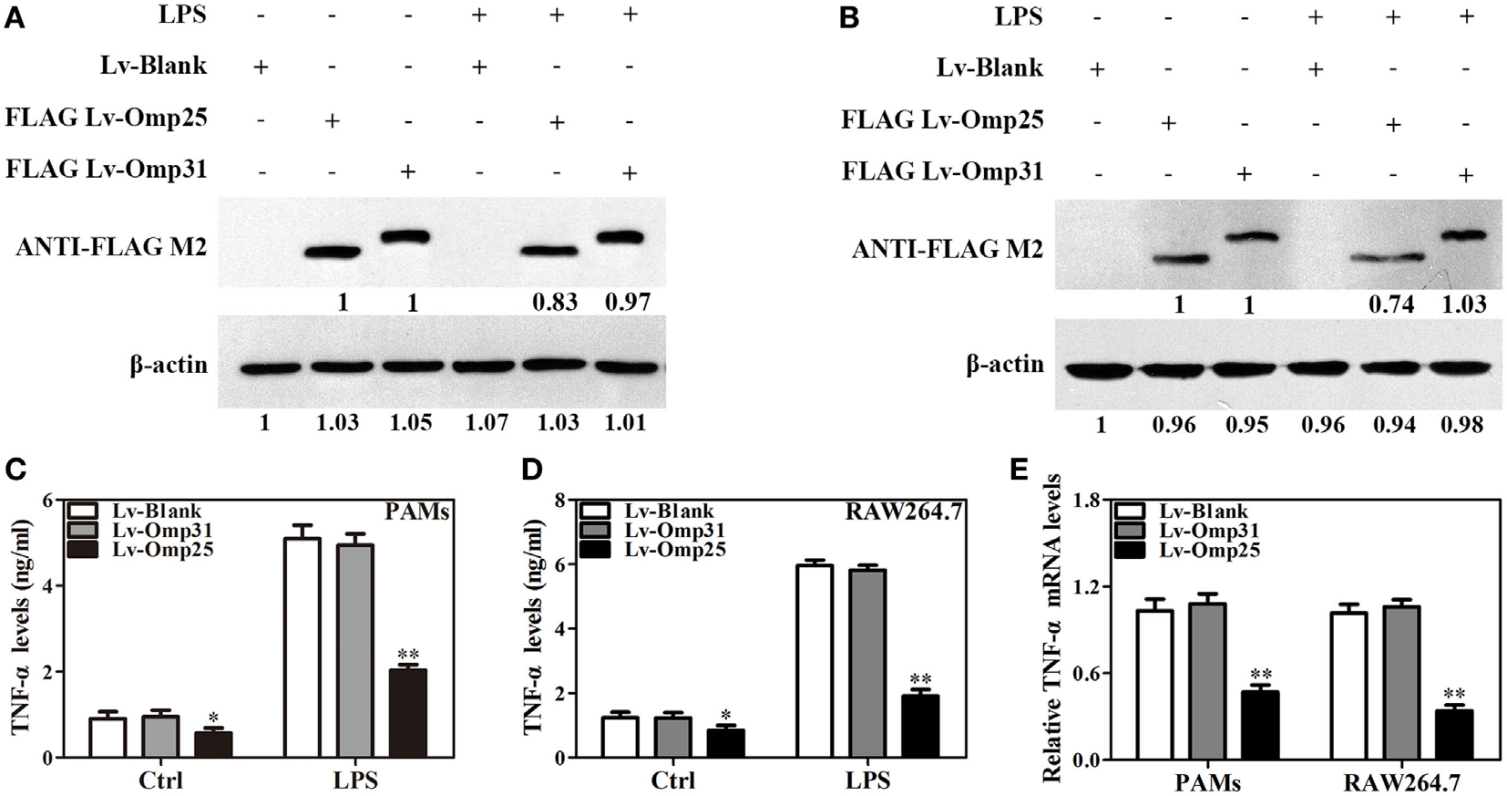
Omp25, but not Omp31, inhibits LPS-induced tumor necrosis factor (TNF)-α production in porcine alveolar macrophages (PAMs) and mouse RAW264.7 cells. **(A,B)** Evaluation of the expression of Omp25 and Omp31 in PAMs and RAW264.7 cells infected with LV-Blank, lentivirus expressing Omp25 (LV-Omp25), or LV-Omp31. PAMs and RAW264.7 cells were, respectively, infected with 100 multiplicities of infection (MOIs) of lentivirus for 24 h, and then treated with or without LPS for 24 h. The expression of protein was detected by western blotting. **(C,D)** Omp25 inhibits LPS-induced TNF-α production in PAMs and RAW264.7 cells. Cells were infected and expression of TNF-α was detected by enzyme-linked immunosorbent assay in culture supernatants. **(E)** Omp25 decreases the levels of TNF-α mRNA in LPS-treated PAMs and RAW264.7 cells. Cells were, respectively, infected with 100 MOIs of lentivirus for 24 h and stimulated with LPS for 6 h, Q-PCR was used to measure the levels of TNF-α mRNA. Values are mean ± SEM of three independent experiments. *P < 0.05, **P < 0.01 versus LV-Blank-infected cells in the same processing.

In order to further examine if Omp25 and Omp31 can directly affect TNF-α production in macrophage, we determined the levels of TNF-α production in PAMs and mouse RAW264.7 cells, following LPS stimulation of the cells infected with lentivirus expressing Omp25 (LV-Omp25), Omp31 (LV-Omp31), or control blank lentivirus (LV-Blank). In LV-Omp25-infected PAMs and RAW264.7 cells, the expression of Omp25 was verified (Figures 2A,B), and TNF-α production was found to be lower than that in LV-Blank-infected cells regardless of LPS stimulation or not (Figures 2C,D). However, in LV-Omp31-infected PAMs and RAW264.7 cells, despite a high level expression of Omp31 (Figures 2A,B), TNF-α production was similar to that in LV-Blank-infected cells (Figures 2C,D). Consistently, the level of TNF-α mRNA in LV-Omp25-infected cells was lower than that in LV-Blank-infected cells, yet the level of TNF-α mRNA in LV-Omp31-infected cells was similar to that in LV-Blank-infected cells (Figure 2E). In addition, we found that Omp25, but not Omp31, also inhibited LPS-induced IL-12 p40 or IL-6 expression in both PAMs and mouse RAW264.7 cells, but did not affect IL-1β expression at both protein and mRNA levels (Figure S3 in Supplementary Material). These data showed that Omp25, but not Omp31, directly inhibited the production of LPS-induced TNF-α, IL-12 p40, or IL-6 expression at both protein and mRNA levels in PAMs and mouse RAW264.7 cells, suggesting that TNF-α production are regulated at both transcriptional and posttranscriptional levels in Omp25-expressing cells.

### Omp25 Downregulates the Transcription of TNF-α by Suppressing the Activation of NF-κB Pathway in PAMs and Mouse RAW264.7 Cells

**Figure 3:**
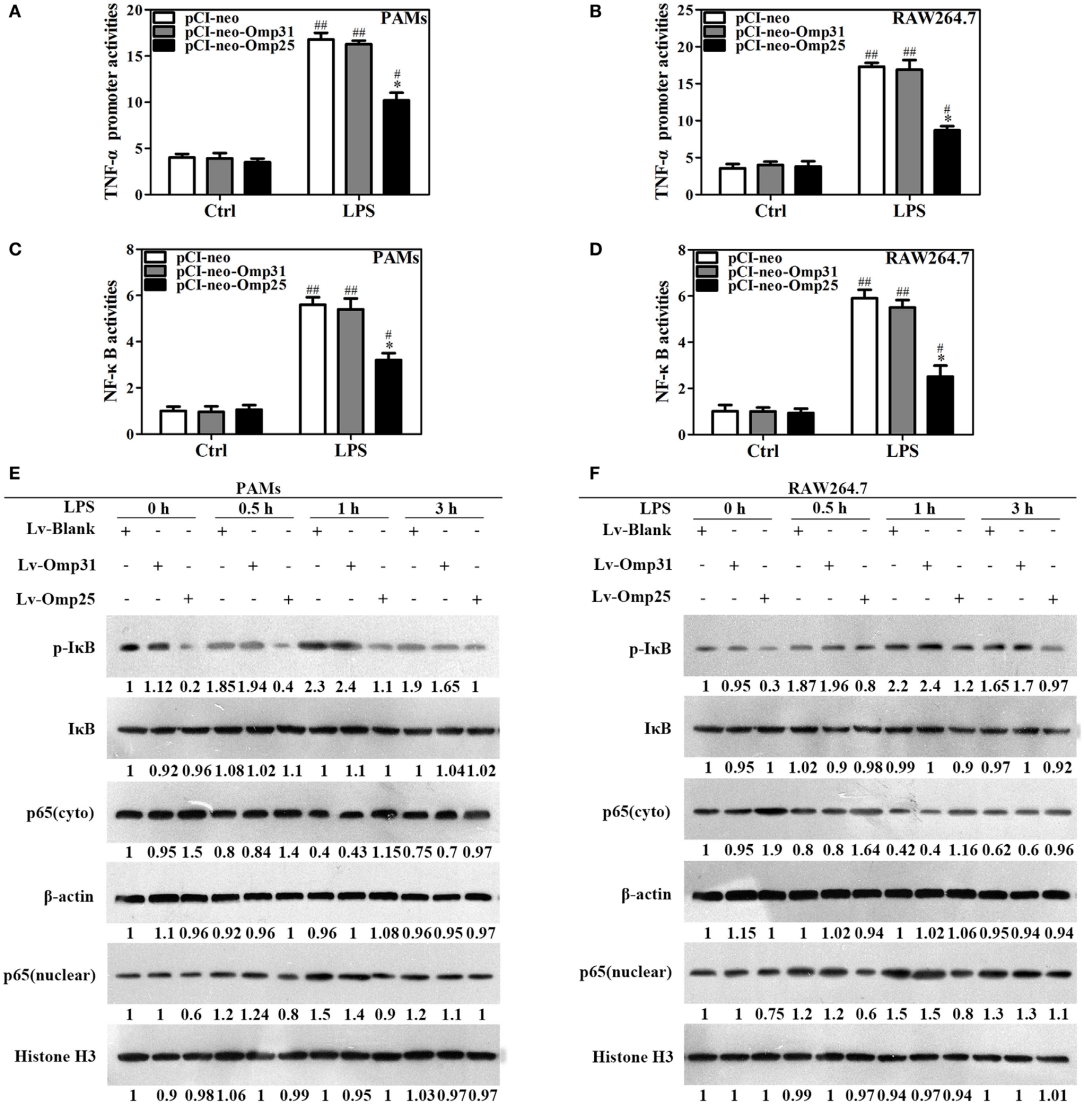
Omp25 inhibits the transcriptional expression of tumor necrosis factor (TNF)-α by suppressing NF-κB pathway activation. **(A–D)** Porcine alveolar macrophages (PAMs) and RAW264.7 cells were, respectively, transfected with pCI-neo, pCI-neo-Omp31, or pCI-neo-Omp25 along with TNF-α or NF-κB luciferase reporter plasmids for 24 h; cells were stimulated with or without LPS for another 24 h, and TNF-α promoter activities **(A,B)** and the relative transcriptional activities of NF-κB **(C,D)** were examined. **(E,F)** PAMs and RAW264.7 cells were infected with 100 multiplicities of infection of LV-Blank, lentivirus expressing Omp25 (LV-Omp25), or LV-Omp31 for 24 h and the expression levels of cytoplasmic p-IκB, IκB, or p65 and nucleoprotein p65 at 0, 0.5, 1, and 3 h following LPS stimulation were determined by western blotting. The results are mean ± SEM of three independent experiments. *P < 0.05 versus LV-Blank-infected cells; ^#^*P* < 0.05, ^##^*P* < 0.01 versus control (Ctrl) for same transfection.

To understand the effects of Omp25 on the TNF-α transcription, we analyzed the activity of TNF-α promoter and NF-κB transcriptional activity using luciferase reporter assays in PAMs and mouse RAW264.7 cells. Upon LPS stimulation, the activities of TNF-α promoter in PAMs and mouse RAW264.7 cells expressing Omp25 were markedly lower than that in the cells expressing Omp31 or control (Figures 3A,B). Similarly, the transcriptional activity of NF-κB was also lower in Omp25-expressing cells than that in Omp31-expressing cells or control plasmid-infected cells (Figures 3C,D). In comparison to LV-Omp31 or LV-blank infection, LV-Omp25 infection resulted in reduced levels of IκB phosphorylation, especially in the first 1 h following LPS stimulation, and also less nuclear NF-κB p65 in both cells (Figures 3E,F). These results suggest that Omp25 inhibits the transcriptional expression of TNF-α *via* suppressing the activation of NF-κB signaling pathway in macrophages.

### Omp25 Upregulates miR-146a, -181a, -181b, and -301a-3p in Both PAMs and Mouse RAW264.7 Cells, but Separately Upregulates miR-130a-3p and miR-351-5p in These Two Cells

**Figure 4:**
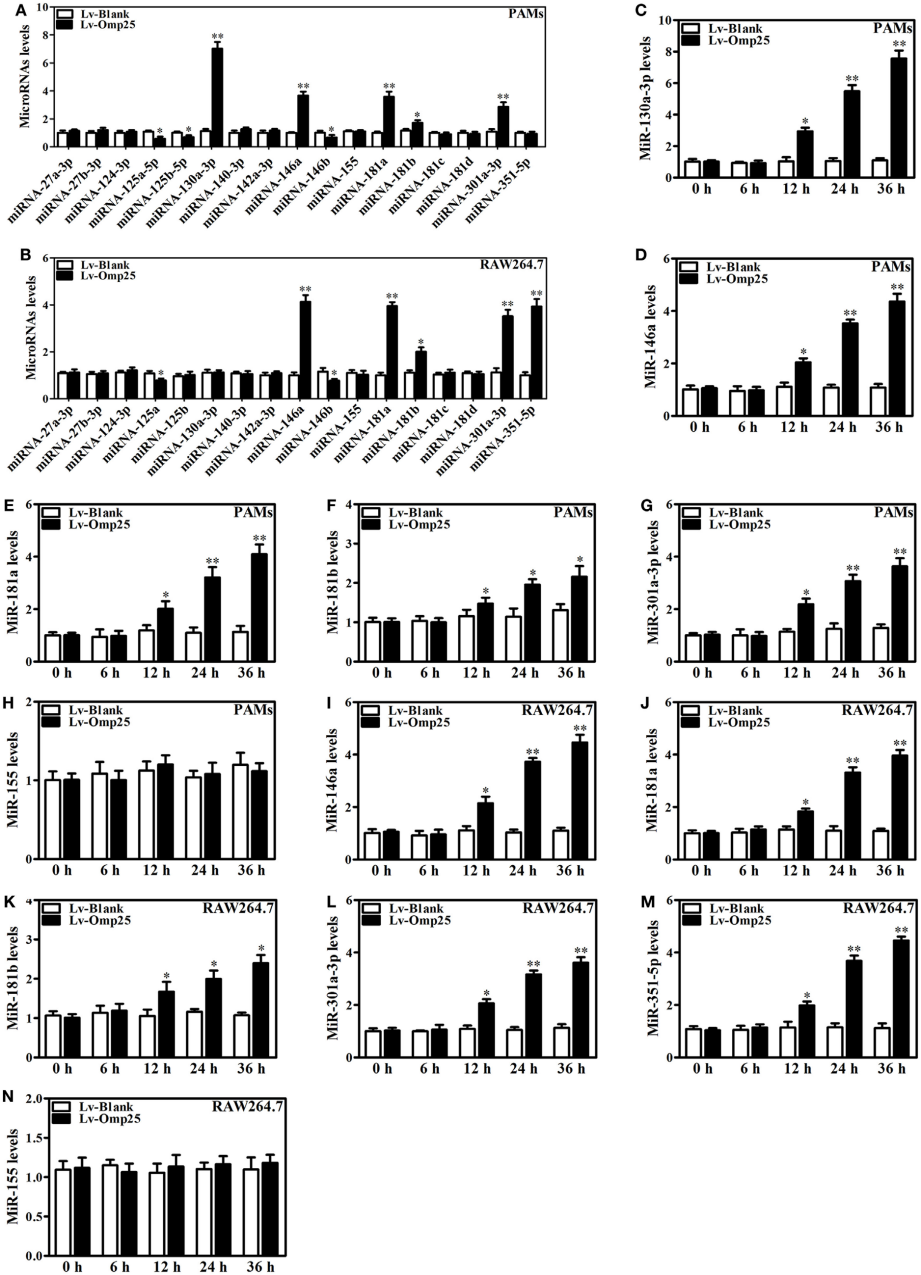
Omp25 upregulates miR-130a-3p, -146a, -181a, -181b, or -301a-3p in porcine alveolar macrophages (PAMs) and miR-146a, -181a, -181b, -301a-3p, or -351-5p in mouse RAW264.7 cells. **(A,B)** Expression profiling of microRNAs in Omp25-expressing PAMs and mouse RAW264.7 cells. Quantitative polymerase chain reaction (Q-PCR) assay was used to measure the levels of 17 specific miRNAs normalized by RNU6B at 24 h following infection. **(C–H)** Q-PCR was used to measure the kinetics of miR-130a-3p, miR-146a, miR-181a, miR-181b, miR-301a-3p, and miR-155 expression in PAMs infected with LV-Blank or lentivirus expressing Omp25 (LV-Omp25). **(I–N)** Q-PCR was used to measure the kinetics of miR-146a, miR-181a, miR-181b, miR-301a-3p, miR-351-5p, and miR-155 expression in mouse RAW264.7 cells infected with LV-Blank or LV-Omp25. Results are mean ± SEM of three independent experiments. *P < 0.05, **P < 0.01 versus LV-Blank-infected cells for same miRNAs or same time point.

miRNAs play important roles in regulating inflammatory mediators and pathogenic infections. To explore the roles of microRNAs in regulation of TNF-α in Omp25-expressing cells, we designed specific primers of the miRNAs targeting TNF-α, TRAF6, or IRAK1 (key regulators of NF-κB pathway) (Figure S4 in Supplementary Material), which were conserved among vertebrates, *via* TargetScan. In PAMs and mouse RAW264.7 cells, we examined the 17 miRNAs expression profiles by Q-PCR assay, some of which have been reported to regulate NF-κB signaling, including miR-146a, miR-146b, miR-155, and miR-351-5p. Our results showed that in Omp25-expressing PAMs, the levels of miR-130a-3p, miR-146a, miR-181a, miR-181b, and miR-301a-3p were upregulated, while miR-125a-5p, miR-125b-5p, and miR-146b were downregulated compared to controls (Figure 4A). In mouse RAW264.7 cells, miR-146a, miR-181a, miR-181b, miR-301a-3p, and miR-351-5p were upregulated, while miR-125a-5p and miR-146b were downregulated (Figure 4B). Considering that miR-130a-3p, miR-146a, miR-181a, miR-181b, miR-301a-3p, and miR-351-5p might participate in the negative regulation of TNF-α production in Omp25-expressing cells, we measured the expression of these miRNAs in PAMs and mouse RAW264.7 cells after LV-Omp25 infection. Results showed that the levels of miR-130a-3p, miR-146a, miR-181a, miR-181b, and miR-301a-3p increased at 12–36 h in LV-Omp25-infected PAMs, whereas miR-130a-3p, miR-146a, miR-181a, miR-181b, miR-301a-3p, and miR-351-5p showed similar changes in LV-Omp25-infected RAW264.7 cells (Figures 4C–G,I–M). Of note, miR-155 did not show significant changes in either PAMs and mouse RAW264.7 cells after LV-Omp25 infection (Figures 4H,N). Altogether, these data demonstrate that Omp25 induces the expression of several miRNAs in PAMs and mouse RAW264.7 cells, with miR-146a, miR-181a, miR-181b, and miR-301a-3p being commonly upregulated in both PAMs and mouse RAW264.7 cells compared to miR-130a-3p and miR-351-5p, which are specific for PAMs and mouse RAW264.7 cells, respectively.

### miR-130a-3p, miR-146a, miR-181a, miR-181b, miR-301a-3p, and miR-351-5p Inhibit TNF-α Expression at Transcriptional or Posttranscriptional Levels

**Figure 5:**
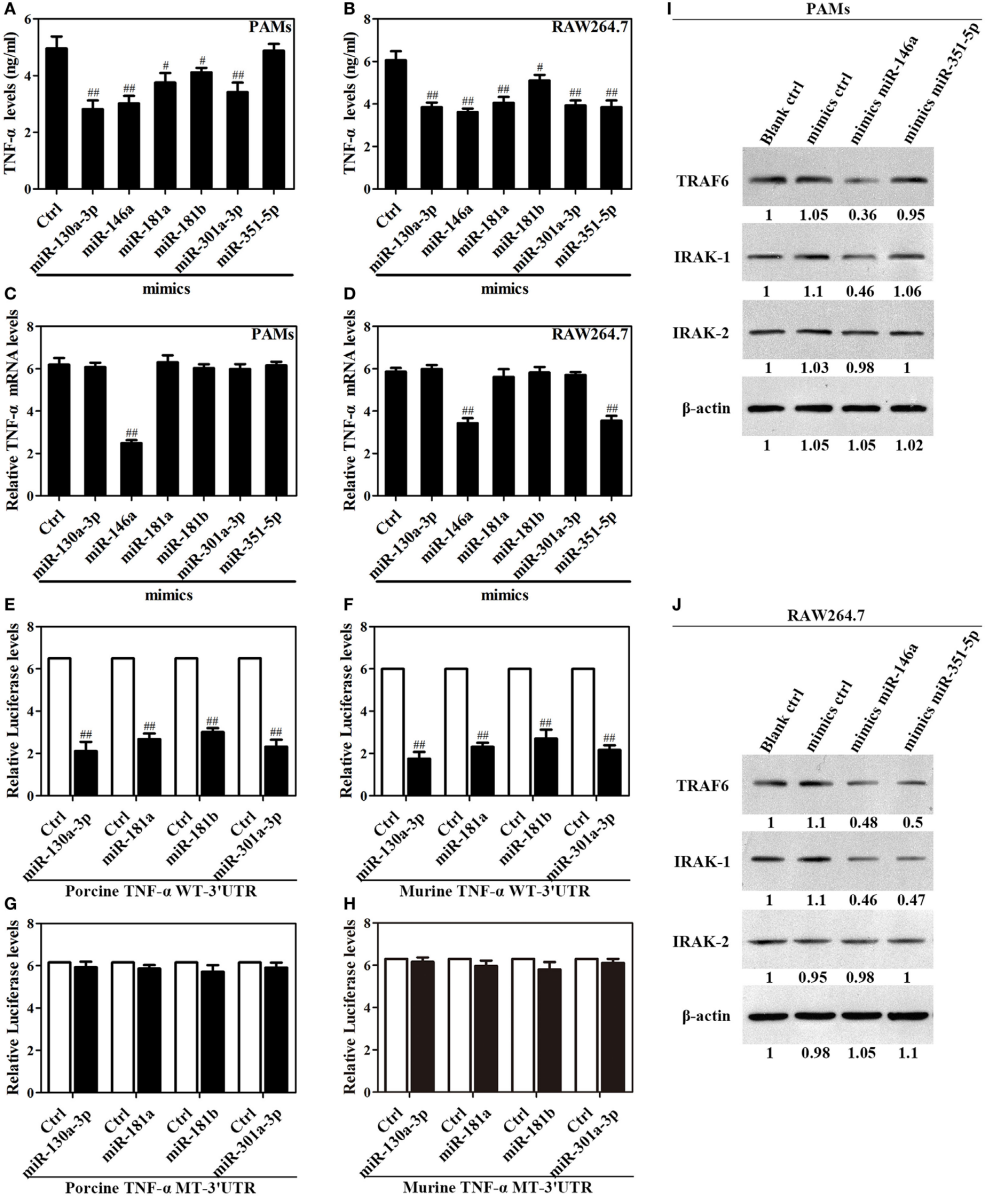
Upregulation of miR-130a-3p, miR-146a, miR-181a, miR-181b, miR-301a-3p, and miR-351-5p blocks LPS-stimulated TNF-α production. **(A–D)** Porcine alveolar macrophages (PAMs) and mouse RAW264.7 cells were transfected with mimics control, or indicated miRNA mimics, and cells were treated with LPS for 24 h, and the levels of TNF-α protein and mRNA were detected by enzyme-linked immunosorbent assay and quantitative polymerase chain reaction, respectively. **(E–H)** HEK-293 cells were transfected with wild-type or mutated TNF-α 3′ untranslated region (UTR) firefly luciferase reporter plasmids along with pTK-Renilla luciferase plasmids, mimics control or different miR-mimics for 48 h, and luciferase activity was measured. **(I,J)** PAMs and RAW264.7 cells were transfected mimics control, or indicated miR-mimics; cells were stimulated with LPS for 24 h, and the levels of TRAF6, IRAK1 and IRAK2 were determined by western blotting. The results are mean ± SEM of three independent experiments. ^#^*P* < 0.05, ^##^*P* < 0.01 versus cells transfected mimics control (Ctrl).

Given above results, we reasoned that miR-130a-3p, miR-146a, miR-181a, miR-181b, miR-301a-3p, and miR-351-5p likely play crucial roles in the Omp25 inhibition of LPS-induced TNF-α production. To test this, PAMs and mouse RAW264.7 cells were transfected with miRNA control, miR-130a-3p mimics, miR-146a mimics, miR-181a mimics, miR-181b mimics, miR-301a-3p mimics, or miR-351-5p mimics, and stimulated the transfected cells with LPS for 24 h. In PAMs, transfection of the mimics of miR-130a-3p, miR-146a, miR-181a, miR-181b, and miR-301a-3p decreased LPS-induced TNF-α, except miR-351-5p (Figure 5A). In RAW264.7 cells, transfection of the mimics of miR-130a-3p, miR-146a, miR-181a, miR-181b, miR-301a-3p, and miR-351-5p decreased LPS-induced TNF-α (Figure 5B). Interestingly, only miR-146a mimics inhibited the level of TNF-α mRNA in PAMs (Figure 5C), whereas both miR-146a and miR-351-5p mimics inhibited TNF-α mRNA level in mouse RAW264.7 cells (Figure 5D). These results suggest that miR-130a-3p, miR-181a, miR-181b, and miR-301a-3p inhibit TNF-α expression at the posttranscriptional level, while miR-146a and miR-351-5p likely inhibit TNF-α expression in transcriptional level.

To confirm that TNF-α is regulated at posttranscriptional level by which miRNA, we constructed reporter plasmids encoding the WT 3′UTR of porcine or murine TNF-α mRNA downstream of the firefly luciferase gene (porcine or murine TNF-α WT-3′UTR), as well as parallel plasmids containing mismatches in the predicted binding sites (miR-130a-3p, miR-181a, miR-181b, miR-301a-3p, or miR-351-5p MT-3′UTR) of the 3′UTR region (Figure S5 in Supplementary Material). Reporter assays showed that miR-130a-3p, miR-181a, miR-181b, and miR-301a-3p decreased the levels of relative luciferase activities from porcine or murine TNF-α WT-3′UTR in contrast to miRNA mimics (Figures 5E,F), whereas luciferase activities did not change in cells transfected with the respective mutated-type reporters (Figures 5G,H). However, miR-351-5p only decreased the level of relative luciferase activity from murine TNF-α WT-3′UTR in contrast to miRNA mimics (Figure S6 in Supplementary Material). These data demonstrate miR-130a-3p, miR-181a, miR-181b, or miR-301a-3p regulates the TNF-α expression at the transcriptional level *via* targeting its 3′UTR, while miR-351-5p may inhibit murine TNF-α expression both at transcriptional and posttranscriptional level.

Next, In order to verify the target molecules of miR-146a and miR-351-5p in porcine cells and murine cells, PAMs and mouse RAW264.7 cells were transfected with mimics control, miR-146a mimics or miR-351-5p mimics, and then stimulated with LPS for 24 h. In PAMs, miR-146a mimics significantly suppressed both TRAF6 and IRAK1 expression while miR-351-5p did not have any effect on either protein (Figure 5I). In mouse RAW264.7 cells, both miR-146a mimics and miR-351-5p mimics showed an obvious inhibitory effect on TRAF6 and IRAK1 (Figure 5J). None of these miRNA mimics showed any effect on IRAK2 in either PAMs or mouse RAW264.7 cells (Figures 5I,J). These results demonstrate that miR-146a and miR-351-5p can repress LPS-induced TNF-α production through targeting same molecules in cells from different species.

### Omp25 Induces miR-146a and miR-351-5p to Inhibit the Transcription of TNF-α *via* Targeting TRAF6 and IRAK1

**Figure 6:**
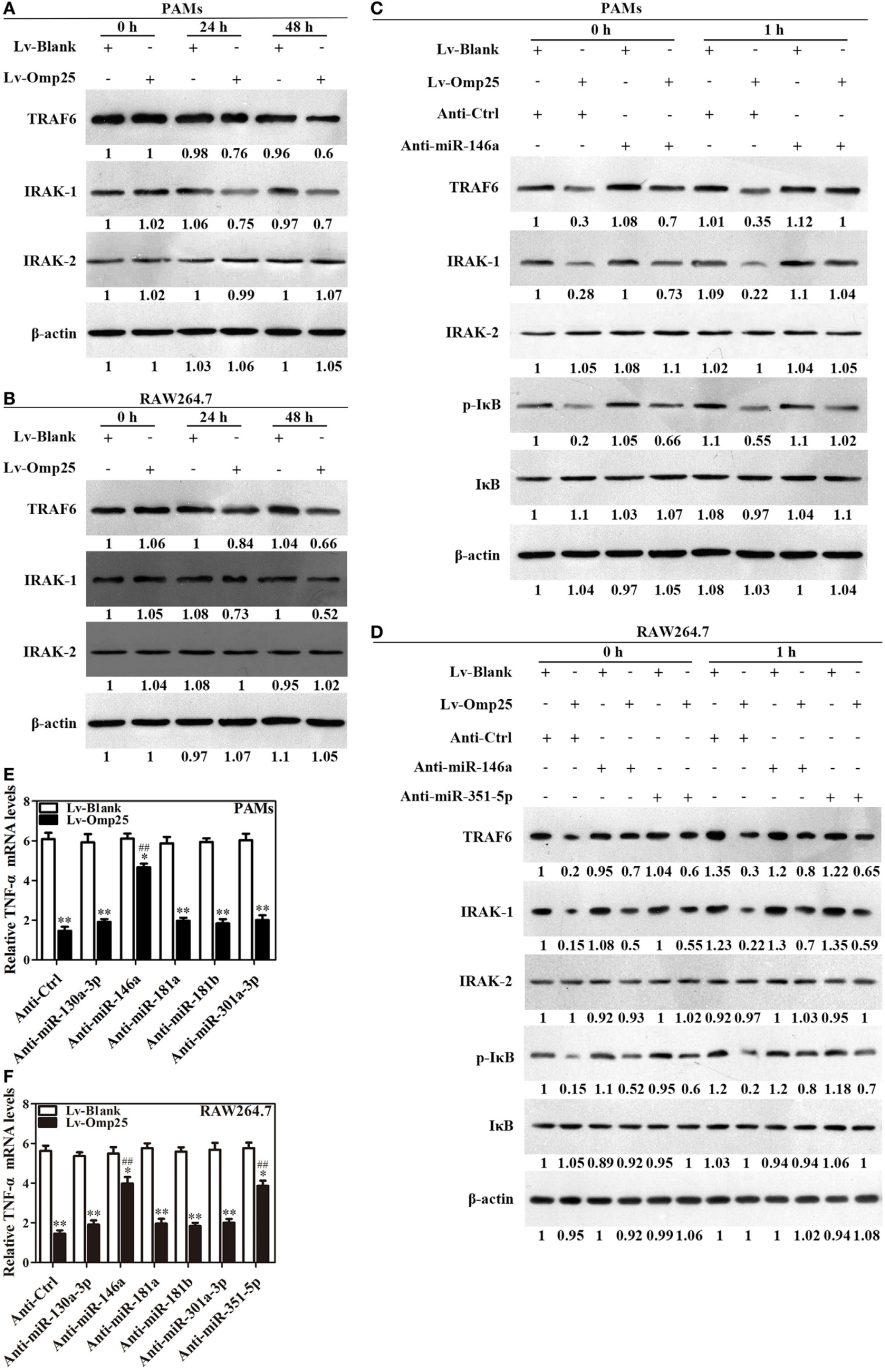
Omp25-induced miR-146a and miR-351-5p inhibit the transcriptional expression of tumor necrosis factor (TNF)-α by targeting to TRAF6 and IRAK1. **(A,B)** Porcine alveolar macrophages (PAMs) and mouse RAW264.7 cells were infected with LV-Blank and lentivirus expressing Omp25 (LV-Omp25), and western blotting was used to determine the expressions of TRAF6, IRAK1, and IRAK2 at 0, 24, and 48 h. **(C,D)** PAMs and mouse RAW264.7 cells were transfected with anti-miRNA control or indicated anti-miRNAs; then, cells were infected with LV-Blank or LV-Omp25 for 24 h, following LPS stimulation for another 1 h, and cells were lysed and examined for TRAF6, IRAK1, IRAK2, p-IκB, and IκB by western blotting. **(E,F)** Cells were transfected with anti-miRNA control or indicated anti-miRNAs and then infected with LV-Blank or LV-Omp25 for 24 h; following LPS treatment for 6 h, quantitative polymerase chain reaction was used to measure the level of TNF-α mRNA. The results are mean ± SEM of three independent experiments. *P < 0.05, **P < 0.01 versus LV-Blank-infected cells; ^##^*P* < 0.01 versus LV-Omp25-infected cells with anti-control (Anti-Ctrl).

Given Omp25 upregulates miR-146a and miR-351-5p, which inhibit TRAF6 and IRAK1 expression in PAMs or mouse RAW264.7 cells, we examined directly whether or not Omp25 expression affects the levels of TRAF6 and IRAK1 in PAMs and mouse RAW264.7 cells. As would be expected, TRAF6 and IRAK1 were decreased in LV-Omp25-infected cells compared to the cells infected with LV-Blank 24 h post-infection, with no change for IRAK2 (Figures 6A,B).

To verify the regulatory roles of miR-146a and miR-351-5p in Omp25 inhibition of TNF-α transcription, PAMs and mouse RAW264.7 cells were, respectively, transfected with an inhibitor control, miR-146a inhibitor, or miR-351-5p inhibitor, and then infected with LV-Blank or LV-Omp25, followed by LPS stimulation for 0 and 1 h. In the presence of miRNA inhibitor control, LV-Omp25 significantly decreased the levels of p-IκB, TRAF6, and IRAK1 in both PAMs and mouse RAW264.7 cells compared to LV-Blank (Figures 6C,D), yet the presence of miR-146a inhibitor significantly reduced the inhibitory effects of LV-Omp25 on these molecules in PAMs (Figure 6C). Similarly, either miR-146a inhibitor or miR-351-5p inhibitor also decreased the inhibitory effects of LV-Omp25 on p-IκB, TRAF6, and IRAK1 in mouse RAW264.7 cells (Figure 6D). These results further confirm that Omp25 inhibits TRAF6 and IRAK1 expression in PAMs and RAW264.7 cells through upregulating miR-146a or miR-351-5p.

We next examined the effects of LV-Omp25 on TNF-α transcription in PAMs and mouse RAW264.7 cells in the presence of these miRNAs inhibitors. Results showed that miR-146a inhibitor markedly decreased the inhibitory effects of Omp25 on TNF-α transcription in PAMs, while both miR-146a and miR-351-5p inhibitors significantly reduced the inhibition of Omp25 on TNF-α transcription in mouse RAW264.7 cells (Figures 6E,F). In addition, we observed miR-351-5p only inhibited the expression of mouse TNF-α at the posttranscriptional level (Figure S6 in Supplementary Material). Above results demonstrate that Omp25 induces miR-146a in PAMs and miR-146a and miR-351-5p in mouse RAW264.7 cells, respectively, which suppresses the transcription of TNF-α *via* targeting TRAF6 and IRAK1 to negatively regulate NF-κB signaling.

### miR-146a, miR-181a, and miR-301a-3p Participate in the Regulation of TNF-α in Both PAMs and Mouse RAW264.7 Cells, whereas miR-130a-3p and miR-351-5p Specially Regulates TNF-α Expression in Porcine and Murine Cells

**Figure 7:**
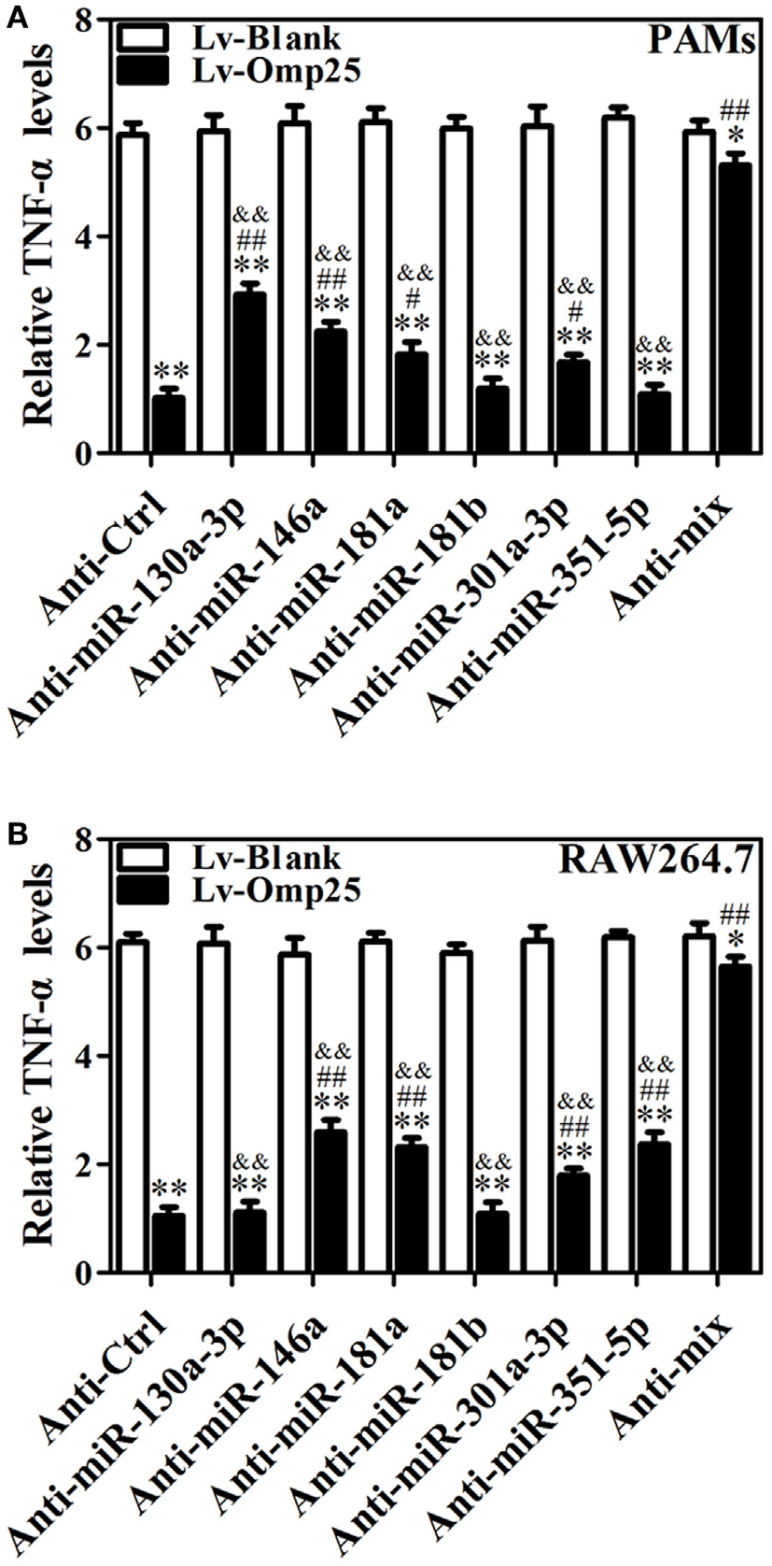
miR-146a, miR-181a, and miR-301a-3p participate in the regulation of tumor necrosis factor (TNF)-α in both porcine alveolar macrophages (PAMs) and mouse RAW264.7 cells, whereas miR-130a-3p and miR-351-5p differentially regulate TNF-α expression in porcine and murine cells. **(A,B)** PAMs and mouse RAW264.7 cells were transfected with anti-control, or indicated anti-miRNA, or anti-miRNAs mix (4 miRNA inhibitors); then, cells were infected with LV-Blank or lentivirus expressing Omp25 (LV-Omp25) for 24 h, and the levels of TNF-α were measured by enzyme-linked immunosorbent assay. The results are mean ± SEM of three independent experiments. *P < 0.05, **P < 0.01 versus LV-Blank-infected cells; ^#^*P* < 0.05, ^##^*P* < 0.01 versus LV-Omp25-infected cells with anti-control (Anti-Ctrl); ^&&^*P* < 0.01 versus LV-Omp25-infected cells with anti-mix (Anti-mix).

To further determine the roles of miR-130a-3p, miR-146a, miR-181a, miR-181b, miR-301a-3p, and miR-351-5p in regulating TNF-α, cells were transfected with inhibitor control, miR-130a-3p, miR-146a, miR-181a, miR-181b, miR-301a-3p, miR-351-5p inhibitor, or miRNA inhibitor mix and infected with LV-Omp25 or LV-Blank. The expression of the respective miRNA was downregulated over threefold by inhibitors, compared with control inhibitor-transfected cells (Figure S7 in Supplementary Material). In PAMs, transfection of miR-130a-3p, miR-146a, miR-181a, and miR-301a-3p inhibitors apparently improved the relative TNF-α levels compared with the inhibitor control, but miR-181b and miR-351-5p inhibitors had no effects on TNF-α expression (Figure 7A). In mouse RAW264.7 cells, except for miR-130a-3p and miR-181b inhibitors, other miRNA inhibitors significantly raised the relative levels of TNF-α (Figure 7B). Notably, in PAMs transfected with a mix of miR-130a-3p, miR-146a, miR-181a, and miR-301a-3p inhibitors, as well as in mouse RAW264.7 cells transfected with a mix of miR-146a, miR-181a, miR-301a-3p, and miR-351-5p inhibitors, the inhibitory effects of Omp25 on TNF-α induction were further attenuated, leading to the levels of TNF-α were almost close to that of LV-Blank (Figures 7A,B). These results demonstrate that in the process of Omp25 inhibiting TNF-α expression, miRNAs (miR-130a-3p, miR-146a, miR-181a, and miR-301a-3p) play principal roles in PAMs, while miRNAs (miR-146a, miR-181a, miR-301a-3p, and miR-351-5p) play principal roles in mouse RAW264.7 cells. Altogether, these results indicate that miR-146a, miR-181a, and miR-301a-3p participate in the regulation of TNF-α in both PAMs and mouse RAW264.7 cells, whereas miR-130a-3p and miR-351-5p specifically regulate TNF-α expression in porcine and murine cells, respectively.

### Downregulation of Omp25-Induced miRNAs Improves TNF-α Production and Promotes Intracellular Bacterial Clearance in WT *B. suis*-Infected Macrophages

**Figure 8:**
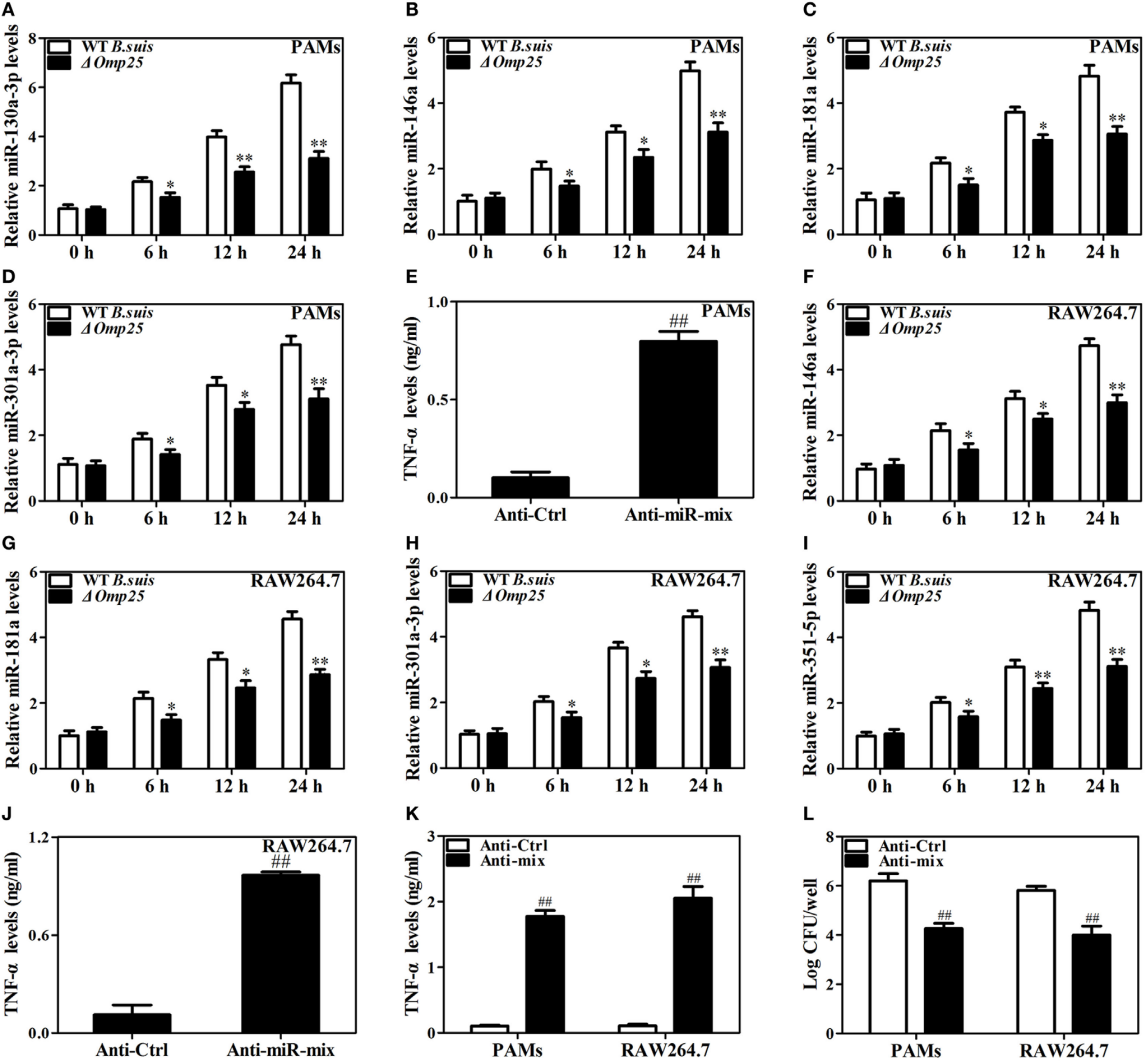
Deficiency of Omp25 decreases *B. suis*-induced miR-130a-3p, miR-146a, miR-181a, miR-301a-3p, or miR-351-5p whereas inhibition of these miRNAs upregulates tumor necrosis factor (TNF)-α and promotes the intracellular clearance of wild-type (WT). *B. suis*. **(A–D)** Porcine alveolar macrophages (PAMs) were infected with WT *B. suis* or Δ*omp25 B. suis* for 0, 6, 12, and 24 h, and quantitative polymerase chain reaction (Q-PCR) was used to analyze the levels of indicated miRNAs. **(E)** PAMs were transfected anti-control or anti-miRNAs mix; cells were infected with WT *B. suis* for 24 h, and TNF-α production was measured by enzyme-linked immunosorbent assay (ELISA). **(F–I)** Mouse RAW264.7 cells were infected with WT *B. suis* or Δ*omp25 B. suis* for 24 h, and cells were harvested to examine the expression of indicated miRNAs by Q-PCR at 0, 6, 12, and 24 h. **(J)** Mouse RAW264.7 cells were treated as in **(E)** and followed by ELISA measurement of TNF-α in culture supernatants. **(K)** Cells were transfected with anti-miRNA control or anti-miRNAs mix and infected with WT *B. suis* for 24 h; following stimulation with LPS for another 24 h, TNF-α production was measured by ELISA. **(L)** Cells were transfected anti-miRNA control or anti-miRNAs mix; cells were infected with WT *B. suis* for 48 h and the numbers of viable intracellular bacteria were determined as described in Section “Materials and Methods.” The results are mean ± SEM of three independent experiments. *P < 0.05, **P < 0.01 versus WT *B. suis*-infected cells at same time point; ^##^*P* < 0.01 versus Anti-Ctrl.

Now we have shown that Omp25 expression can induce miR-130a-3p, miR-146a, miR-181a, miR-301a-3p, or miR-351-5p in PAMs and mouse RAW264.7 cells, but whether or not the WT *B. suis* infection can also upregulate the expression of these miRNAs needs to be examined. We found that WT *B. suis* induced higher levels of miR-130a-3p, miR-146a, miR-181a, and miR-301a-3p expression than that of Δ*omp25 B. suis* in PAMs (Figures 8A–D). Pretreatment with a mix of these miRNAs inhibitors (Anti-miR-mix) significantly increased TNF-α production in PAMs upon WT *B. suis* infection (Figure 8E). Similarly, in mouse RAW264.7 cells, the levels of miR-146a, miR-181a, miR-301a-3p, and miR-351-5p were higher in WT *B. suis* compared to that infected by Δ*omp25 B. suis* (Figures 8F–I). Pretreatment with a mix of these miRNAs inhibitors (Anti-miR-mix) also significantly increased TNF-α production of mouse RAW264.7 cells upon WT *B. suis* infection (Figure 8J). Moreover, treatment with a mix of these Anti-miRNAs significantly increased TNF-α production in WT *B. suis*-infected PAMs and mouse RAW264.7 cells upon LPS stimulation (Figure 8K). Consequently, pretreatment of cells with a mix of these Anti-miRNAs significantly decreased the intracellular bacterial number of PAMs or mouse RAW264.7 cells upon WT *B. suis* infection (Figure 8L). These results further confirm that Omp25 plays a key role in inhibition of TNF-α production in *Brucella*-infected macrophages, and that blocking of Omp25-induced miRNAs can improve TNF-α production and promote intracellular bacterial clearance in *B. suis*-infected macrophages.

## Discussion

**Figure 9:**
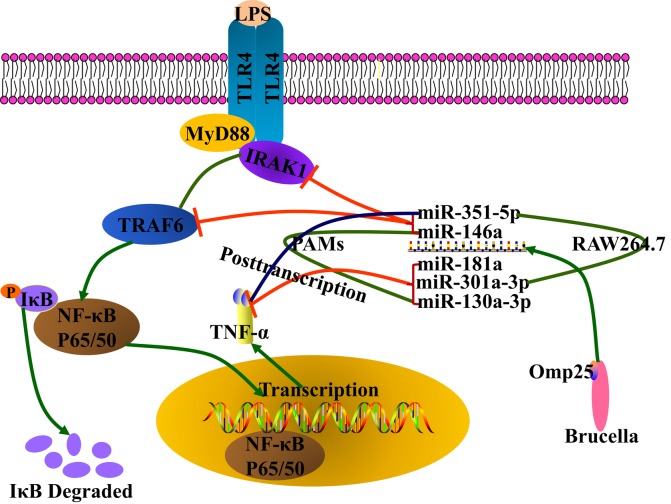
Model for *Brucella* Omp25 modulation of tumor necrosis factor (TNF)-α suppression in porcine and murine macrophages. *Brucella* Omp25 inhibits LPS-induced TNF-α at the transcriptional levels *via* miR-146a (in both porcine and murine macrophages), or miR-351-5p (in murine macrophages) by targeting TRAF6 or IRAK1 to inhibit the activation of NF-κB signaling pathway. At the posttranscriptional levels, *Brucella* Omp25 inhibits LPS-induced TNF-α *via* upregulating miR-181a and miR-301a-3p (in both porcine and murine macrophages), or miR-130a-3p (in porcine macrophages), or miR-351-5p (in murine macrophages).

MicroRNAs are a class of small non-coding RNAs with 19–23 nucleotides in length that interact with target mRNAs by forming incomplete base pairing to regulate the expression of target mRNAs by inhibiting translation or destabilizing mRNAs (28, 29). Mounting evidences have showed that miRNA plays important roles in the regulation of immune response to different infection (30). During *B. melitensis* infection, microRNA expression profiling has identified 57 differential expression miRNAs, which are partially involved in altering the activities of *Brucella*-infected RAW264.7 cells (31). In this study, we found that *B. suis* Omp25 upregulated miR-130a-3p, miR-146a, miR-181a, miR-301a-3p, or miR-351-5p, which was correlated with TNF-α inhibition. Further studies revealed that miR-130a-3p, miR-181a, and miR-301a-3p target to the 3′UTR region of TNF-α to restrain TNF-α production at the posttranscriptional level, whereas miR-146a and miR-351-5p transcriptionally suppress the expression of TNF-α by targeting TRAF6 and IRAK1 (Figure 9). Importantly, *B. suis* infection induces higher levels expression of miR-146a, miR-181a, miR-181b, or miR-301a-3p in both PAMs and mouse RAW264.7 cells, yet specifically upregulates miR-130a-3p in PAMs and miR-351-5p in RAW264.7 cells, respectively. Consistent with these findings, inhibition of these miRNAs altogether attenuates the ability of Omp25 to inhibit LPS-induced TNF-α, resulting in increased TNF-α production and intracellular bacteria clearance in both cells.

Previous studies have reported that *Brucella* infection causes dysregulation of human macrophages and dendritic cells (8, 18). WT *Brucella* infection exhibits a relatively low ability to induce TNF-α expression in lymph nodes and spleens *in vivo*, as well as in the cultured monocytes and dendritic cells *in vitro* (32–34). Whereas Omp25-deficient *B. suis* shows an enhanced ability to induce TNF-α in human macrophages, suggesting that Omp25 is involved in the regulation of TNF-α expression in *B. suis-*infected macrophages (8, 18). However, Omp31, another virulence-related factor, does not appear to regulate TNF-α expression in *B. suis-*infected macrophages (18). The results present in this study further showed that Omp25-deficient *B. suis* induced more TNF-α production and exhibited a lower level of survival compared to WT *B. suis*, whereas Omp31-deficient *B. suis* did not show any difference from WT *B. suis* in TNF-α production and intracellular survival of *B. suis* in PAMs and mouse RAW264.7 cells. Moreover, using lentivirus to express Omp25, directly inhibited the production of LPS-induced TNF-α expression at both protein and mRNA levels in PAMs and mouse RAW264.7 cells, but Omp31 expression did not affect LPS-induced TNF-α expression at both cells. These data further confirm that Omp25, but not Omp31, is involved in the regulation of TNF-α expression in *B. suis-*infected macrophages. Omp31 is another relatively conserved molecule in *Brucella* spp. *B. suis* Omp31 shows 99% identity with *B. melitensis* Omp31. Interestingly, however, *B. melitensis* Omp31 has be reported to be involved in regulation of TNF-α expression in *B. melitensis-*infected macrophages (35), which is only one report about Omp31 regulation of TNF-α up to date. Omp31 shows a relatively lower identity than Omp25 among *Brucella* species, it is worth to further study whether the Omp31 of some *Brucella* species are also involved in regulation of TNF-α. Besides TNF-α, we found that Omp25 also inhibits LPS-induced IL-12 p40 and IL-6 expression in PAMs and mouse RAW264.7 cells, but does not affect IL-1β expression. TNF-α, IL-12 p40, and IL-6 expression are regulated by NF-κB signaling pathway, which has been identified to be involved in *B. suis* Omp25-induced immunosuppression in our previous and current studies, whereas IL-1β is mainly regulated by inflammasome-dependent signaling. These data further indicate that Omp25 is implicated in the regulation of NF-κB signaling but not in inflammasome-dependent signaling during *Brucella* infection.

The NF-κB signaling pathway has been implicated in the transcriptional regulation of many pro-inflammatory cytokines, including TNF-α, IL-12p40, and IL-6 in human macrophages and mouse macrophages (36, 37). Previous studies have showed that miR-146a is rapidly induced in human monocytic THP-1 cells in response to inflammatory stimulation, and pro-inflammatory cytokines have also been identified to modulate innate immune signaling *via* targeting TRAF6 and IRAK1 to represses the miR-146a/b-TRAF6/IRAK1/IRAK2-NF-κB axis (38, 39). In human monocytes and macrophages, miR-146a does not appear to be upregulated by *B. suis* Omp25 or WT *B. suis* infection in our previous study (40). However, in this study, miR-146a was significantly upregulated by Omp25 or WT *B. suis* infection in both PAMs and mouse RAW264.7 cells, which suppresses the transcription of TNF-α *via* targeting TRAF6 and IRAK1. Beside miR-146a, miR-351-5p has also been reported to target TRAF6 to inhibit denervation-induced muscle atrophy (41). In our work, miR-351-5p was found to be upregulated by Omp25 in RAW264.7 cells to inhibit TNF-α transcription *via* targeting TRAF6 and IRAK1, yet in PAMs miR-351-5p was not upregulated by Omp25 to regulate TRAF6 and IRAK1. In addition, miR-155 is known to play a negative regulation role in fine-tuning the inflammation responses mediated by NF-κB and activator protein-1 pathway (39). In our previous study, we also showed that miR-155 is upregulated to negatively regulate IL-12 p40 transcription *via* targeting Tab 2 in Omp25-expressing human monocytes and macrophages (40). However, in this work, we did not detect miR-155 induction in Omp25-expressing or WT *B. suis*-infected PAMs or mouse RAW264.7 cells. These findings further demonstrate that *Brucella* Omp25 can disturb the transcription of pro-inflammatory cytokines in macrophages of different mammals, which are mainly dependent on modulating the activation of NF-κB signaling pathway through different miRNAs that target different signaling molecules in different mammalian cells.

In addition to transcriptional regulation, Omp25 can also directly regulate pro-inflammatory cytokines expression at posttranscriptional level through targeting the 3′UTR of these cytokine mRNAs. Previously, we have identified Omp25 induction of miR-21-5p and miR-23b by directly suppressing LPS/R848-induced IL-12 expression through targeting *il12A* 3′UTR and *il12B* 3′UTR, respectively (40). Herein, we further identified miR-181a and miR-301a-3p to be involved in the posttranscriptional regulation of TNF-α in both Omp25-expressed PAMs and mouse RAW264.7 cells, yet found that Omp25 specially induced miR-130a-3p to inhibit TNF-α expression in porcine macrophages, and Omp25 specially induced miR-351-5p to inhibit TNF-α expression in murine macrophages *via* targeting the 3′UTR of TNF-α. In both PAMs and mouse RAW264.7 cells, we showed that miR-181a and -301a-3p participated in the posttranscriptional regulation of TNF-α inhibition by Omp25, suggesting that *Brucella* Omp25 adopts similar mechanisms to interfere with the expression of TNF-α in macrophages from different mammals through employing these miRNAs. As members of miR-130/301 family, miR-130a and miR-301a have been shown to be modulated by distinct transcriptional events (42) and are highly conserved for the 3′UTR of TNF-α among vertebrate as we observed that miR-130a-3p and -301a-3p mimics could downregulate TNF-α protein levels and reporter gene levels in both PAMs and RAW264.7 cells. miR-130a has been reported to downregulate the expression of TNF-α, thereby inhibiting the activation of macrophage (26, 43). Although miR-130a-3p can target the 3′UTR of TNF-α derived from different mammals, miR-130a-3p inhibitor only could attenuate the inhibitory effect of Omp25 in PAMs since miR-130a-3p was only induced in Omp25-expressed PAMs, but not in RAW264.7 cells. Moreover, we found some differences between ssc-miR-130a (miRBase accession no. MI0008217) and mmu-miR-130a (miRBase accession no. MI0000156) in their precursor sequences and putative promoter sequences. Comparing 2,000 bases region (13,38,972–13,36,973) at the upstream of ssc-miR-130a precursor from Sscrofa 10.2 (GenBank accession no. NW_003609527) genomic DNA with the 2,000 bases region (847,39,116–847,41,115) at the upstream of mmu-miR-130a precursor from Mus musculus (GenBank accession no. NT_039207.8) genomic DNA showed 68% identity by Clustal W method, while the 1,000 nucleotide sequences (13,37,972–13,36,973) at the upstream of ssc-miR-130a only showed 56.2% identity with the 1,000 bases region (847,40,116–847,41,115) at the upstream of mmu-miR-130a precursor. The low homology of miR-130a putative promoter sequences between porcine and murine may cause significant difference in miR-130a expression in Omp25-expressed PAMs and RAW264.7 cells. However, miR-301a-3p could attenuate the inhibition of Omp25 in both PAMs and RAW264.7 cells since miR-301a-3p was induced by Omp25 in both cells. Additionally, miR-181 family also plays crucial roles in inflammation (44), likely by binding to human TNF-α mRNA 3′UTR to promote the fine-tuning of TNF-α in immunoparalysis (45). Taken together, the results present in here demonstrate that miR-130a-3p, miR-181a, and miR-301a-3p regulate TNF-α expression at posttranscriptional level, miR-146a regulates TNF-α expression at posttranscriptional level, whereas miR-351-5p specifically regulate mouse TNF-α expression at both transcriptional and posttranscriptional levels.

Now that miRNAs are involved in the immunosuppression caused by *Brucella*, so whether we can control the occurance of Brucellosis by targeting miRNAs. In this study, we found that the inhibition of miR-130a-3p, -181a, and -301a-3p partially blocked Om25-induced TNF-α inhibition, while inhibition of miR-146a or miR-351-5p attenuates the inhibitory effects of Omp25 or WT *B. suis* on TNF-α transcription. These results not only further confirm that the inhibitory effect of miR-130a-3p, miR-181a, or miR-301a-3p on TNF-α is at posttranscriptional level, likely by targeting to the 3′UTR of TNF-α, but also demonstrate that *Brucella* Omp25 regulates TNF-α expression by multiple modes of action. Since the synergistic regulation of these Omp25-induced miRNAs in TNF-α production, simultaneous depletion of miR-130a-3p, -146a, -181a, and -301a-3p could almost completely block the inhibitory effects of Omp25 on LPS-induced TNF-α in PAMs infected with WT *B. suis*, and simultaneous depletion of miR-146a, -181a, -301a-3p, and -351-5p in mouse RAW264.7 cells could almost completely block Omp25 inhibition, thus promoting LPS-induced TNF-α and increasing intracellular clearance of *Brucella* in both cells. These data demonstrate that inhibition of Omp25-induced miRNAs (Note: all of miRNAs in each species) can efficiently promote TNF-α production and improve the ability to anti-*Brucella* infection of macrophages. Consistently, we also observed that Omp25-deficient *B. suis* showed a lower intracellular survival relative to WT *B. suis* at the 48 h post-infection (not at 24 h, p.i.), suggesting that Omp25 and Omp25-induced miRNAs are correlated with the intracellular survival of *Brucella* and present a certain feature of action. These findings provide basis for targeting of miRNA to control *Brucella* infection and to improve the immunosuppression of Brucellosis in future.

In summary, the present data in this study provide certain evidences for miRNAs (miR-130a-3p, miR-146a, miR-181a, miR-301a-3p, and miR-351-5p) participation of *Brucella* Omp25-induced TNF-α suppression in porcine and murine macrophages and demonstrate that different regulation patterns are employed by Omp25 between porcine and murine macrophages in this regulatory process. Further deeply understanding of the regulation mechanisms of miRNA in *Brucella*-infected macrophage may help to design more effective therapies to improve the outcome of Brucellosis.

## Author Contributions

YH, DT, S-LL, and XL designed the experiments and wrote the paper. XL performed the experiments with the assistance of XJZ, XW, XY, CH, ZW, and QD, XMZ. YH and DT analyzed the data. DT and YH contributed to reagents/materials/analysis tools.

## Conflict of Interest Statement

The authors declare that the research was conducted in the absence of any commercial or financial relationships that could be construed as a potential conflict of interest. The reviewer VG and handling editor declared their shared affiliation.
