# Supplementary material for: Brucella Downregulates Tumor Necrosis Factor-α to Promote Intracellular Survival via Omp25 Regulation of Different MicroRNAs in Porcine and Murine Macrophages
Source: Front Immunol. 2018 Jan 17;8:2013. doi: 10.3389/fimmu.2017.02013 (PMC5776175; doi:10.3389/fimmu.2017.02013)
Supplement: Supplementary file 1 [file data_sheet_1.doc]

Supplementary Material


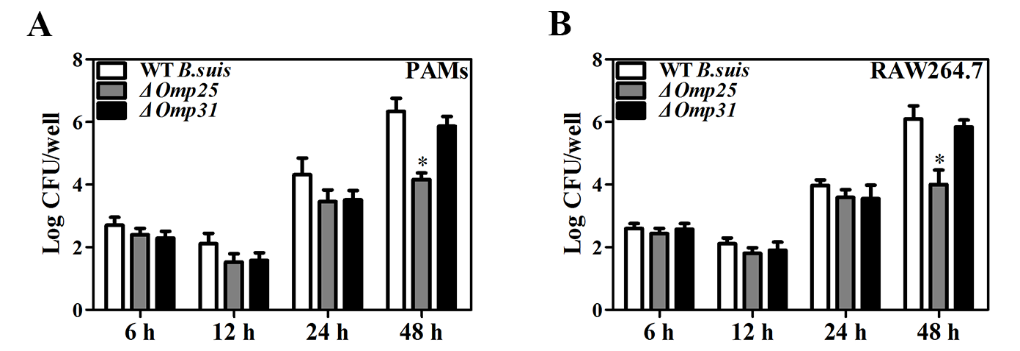


**Fig S1. The intracellular survival of *Δomp25 B. suis* and *Δomp31 B. suis* within PAMs and mouse RAW264.7 cells compared to that of WT *B. suis*. (A, B)** PAMs and mouse RAW264.7 cells were infected with *Δomp25 B. suis* and *Δomp31 B. suis*, WT *B. suis* performed as control. Cells were lysed and the numbers of viable bacteria were then determined at 6 h, 12 h, 24 h and 48 h post infection. The results are means ± SD, representative of three independent experiments. *^*^P* <0.05 versus wild-type *B. suis*-infected cells.

**
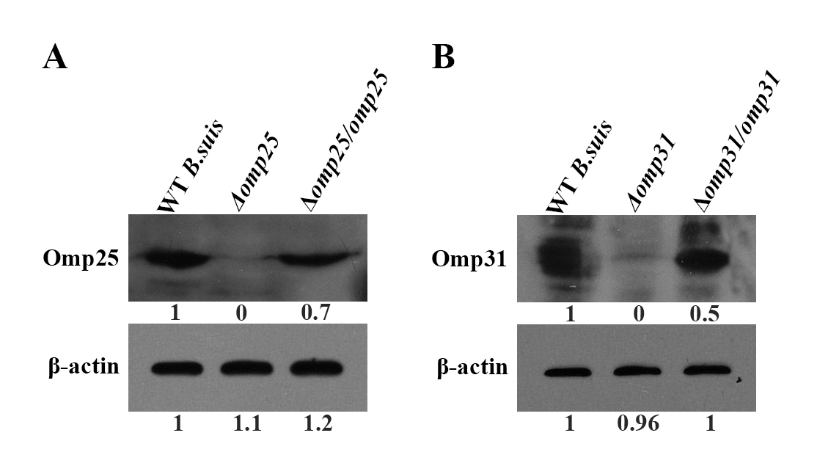
**

**Fig S2. Expression of Omp25 and Omp31 in different *B. suis* infected cells.** **(A)** PAMs were infected with WT *B. suis*, Omp25-deficient mutant (*Δomp25*) and *Δomp25 B. suis* trans complemented with native *omp25* gene (*Δomp25/omp25*) for 24 h, and then analyzed the levels of Omp25 by western blotting. **(B)** PAMs were infected with WT *B. suis*, Omp31-deficient mutant (*Δomp31*) and *Δomp31 B. suis* trans complemented with native *omp31* gene (*Δomp31/omp31*) for 24 h, and then analyzed the levels of Omp31 by western blotting.

**
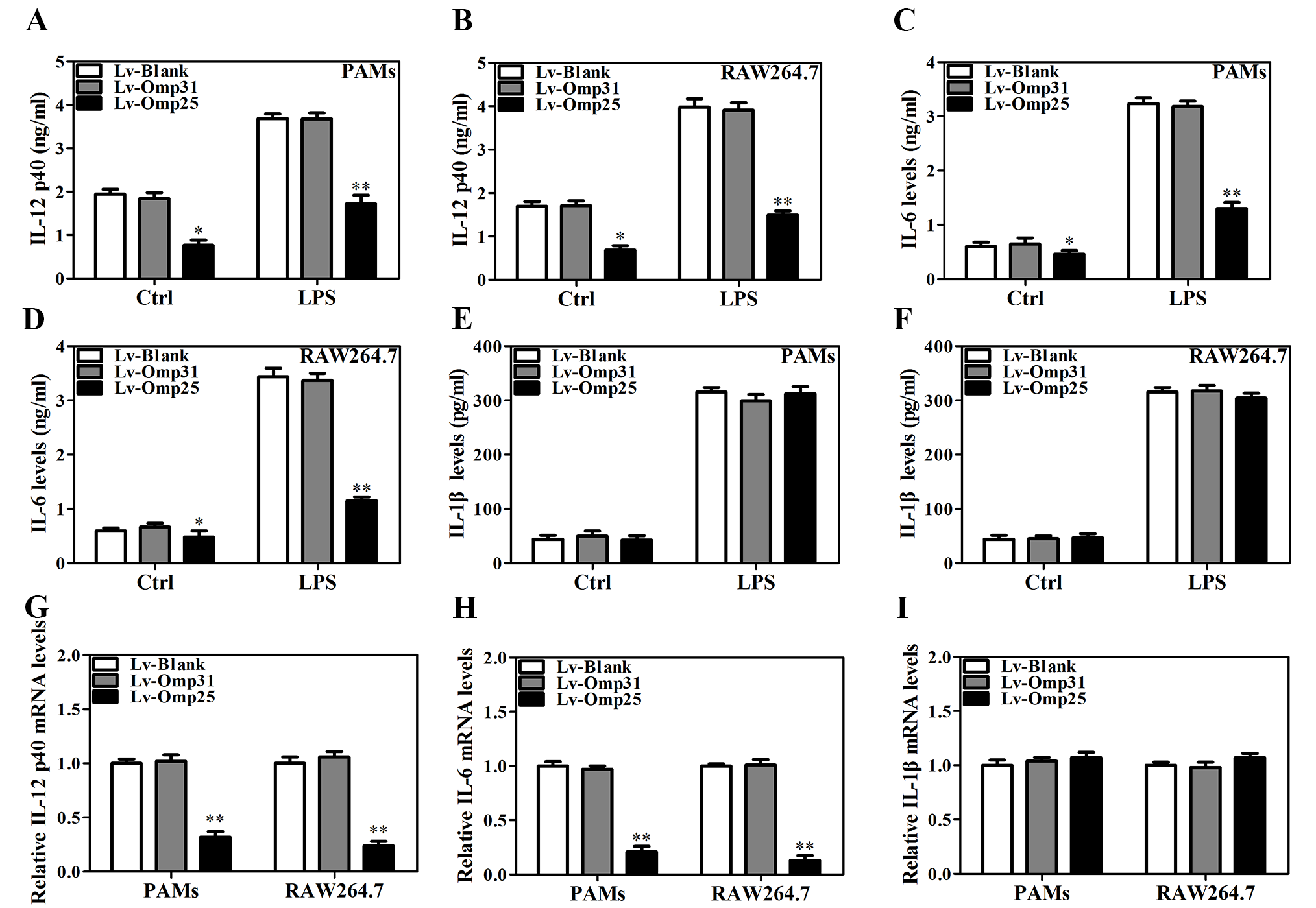
Fig S3. The effects of Omp25 and Omp31 on LPS-induced IL-12 p40, IL-6 or IL-1β expression.** PAMs and RAW264.7 cells were respectively infected with 100 MOIs of lentiviral for 24 h, and then treated with or without LPS for 24 h. The levels of IL-12 p40, IL-6 or IL-1β were detected by ELISA in culture supernatants (A-F). In addition, cells were infected with 100 MOIs of lentivirus for 24 h and stimulated with LPS for 6 h. Q-PCR was used to measure the levels of IL-12 p40, IL-6 or IL-1β mRNA (G-I). The results are means ± SEMs of 3 independent experiments. **P*< 0.05, ***P*< 0.01 versus LV-Blank-infected cells in the same processing.


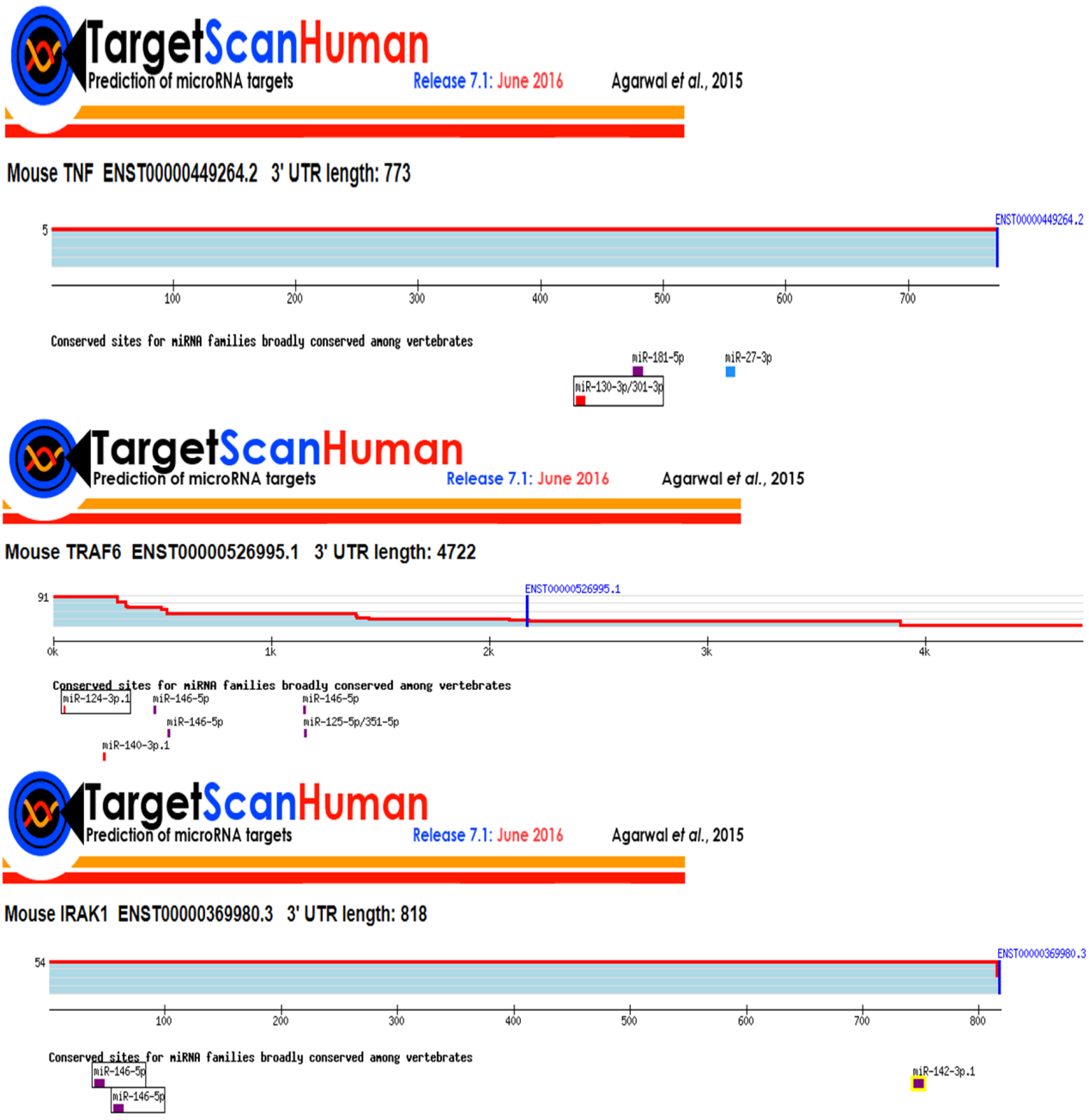


**Fig S4. Predicted miRNA recognition elements in the 3’ UTR regions of TNF-α, TRAF6 and IRAK1 genes by TargetScan.** MiRNA prediction algorithms were used to evaluate the likelihood of miRNA binding to the TNF-α, TRAF6 or IRAK1 3’ UTR.

**
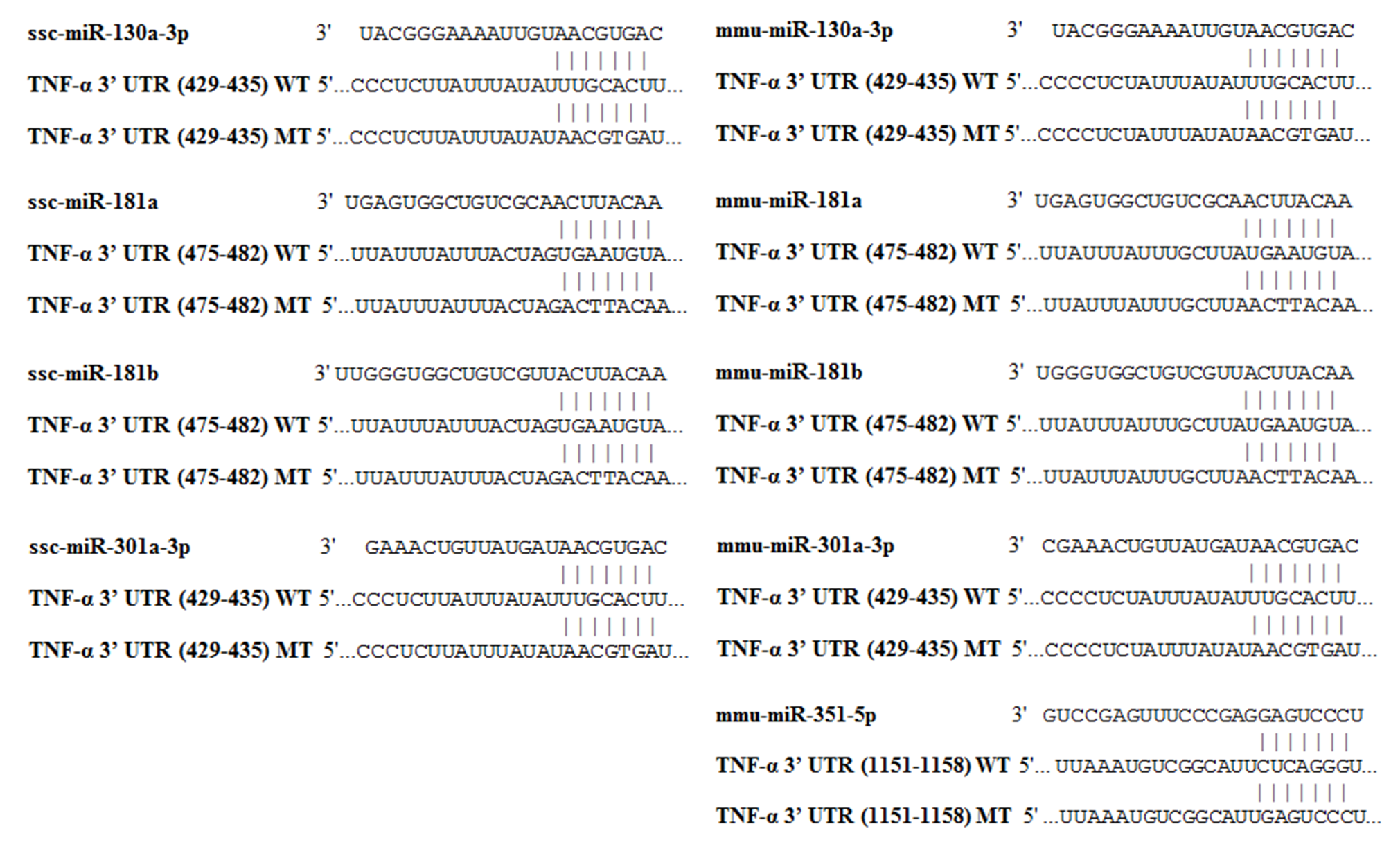
Fig S5. Designed 3’ UTR regions of TNF-α genes sequence with wild-type and mutant type.** The diagram shows a series of sequence pairing of four miRNAs and their target sites in the wild-type 3’UTR regions of TNF-α genes. The mutation sequences for binding sites of the 3’UTR region were also showed.


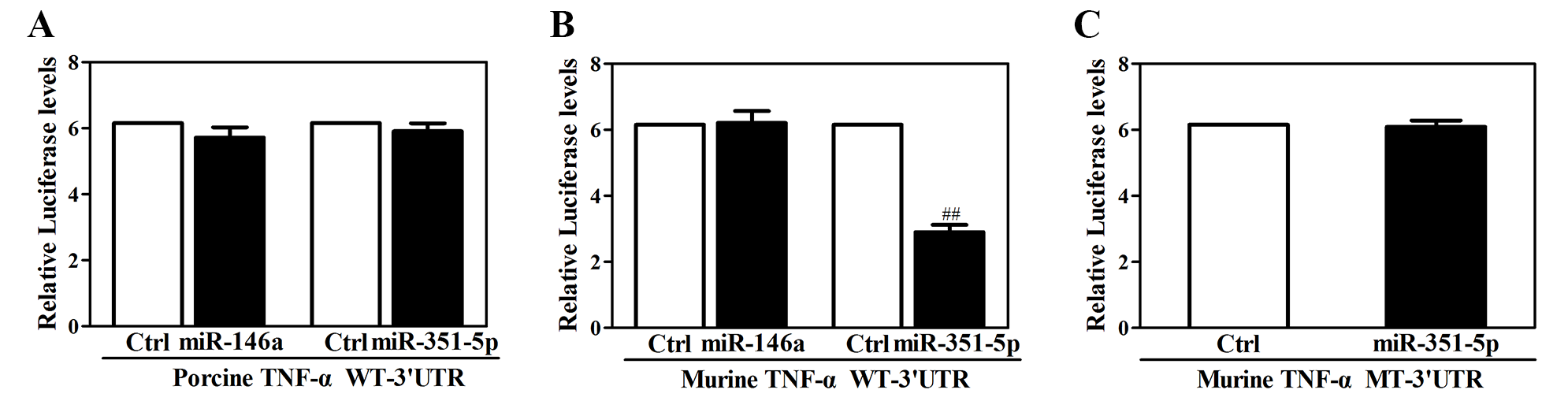


**Fig S6. MiR-351-5p inhibits the expression of mouse TNF-α at the post-transcriptional level.** MiRNAs reduced luciferase activity in cells transfected with wild-type reporter (only murine TNF-α WT-3’UTR), but not in cells transfected with mutated-type reporter (murine TNF-α MT-3’UTR). HEK-293 cells were transfected with wild type (porcine and murine) or mutated type (murine) TNF-α 3’UTR firefly luciferase reporter plasmids, pTK-Renilla-luciferase plasmids, together with mimics control (Ctrl), miR-146a and miR-351-5p mimics. After 48 hours, firefly luciferase activity was measured and normalized by renilla luciferase activity. The results are means ± SD, representative of three independent experiments. ^##^*P* < 0.01 versus cells transfected mimics control (Ctrl).


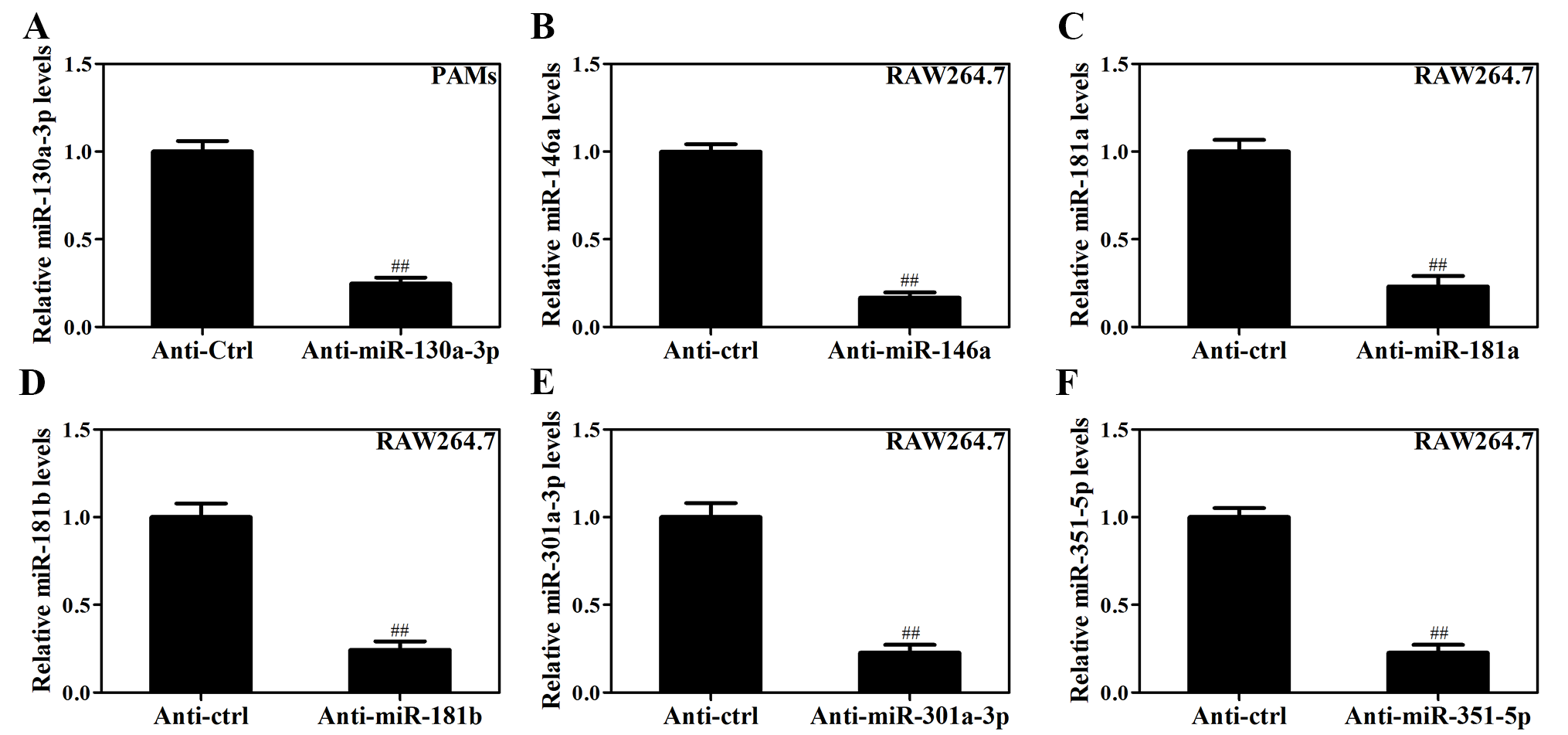


**Fig S7. The effects of miRNA inhibitors.** The LV-Omp25-infected PAMs or RAW264.7 cells were respectively treated with ctrl, miR-130a-3p, miR-146a, miR-181a, miR-181b, miR-301a-3p or miR-351-5p inhibitors for 24 h, and the corresponding miRNAs were measured by Q-PCR and normalized to RNU6B in each sample. The results are means ± SD, representative of three independent experiments. ^##^*P* < 0.01 versus cells transfected inhibitor control.

**TABLE S1 | Sequences of the primers used in this study.**

| **Primer ID**  omp25 FW | **Primer sequence (5’-3’)**  CCGGAATTCATGCGCACTCTTAAGTCTCTC |
| --- | --- |
| Flag-omp25 RW | CGCGGATCCTTACTTATCGTCGTCATCCTTGTAATCGAACTTGTAGCCGATGCC |
| omp31 FW | CCG GAATTCATGAAATCCGTAATTTTGGC |
| Flag-omp31 RW | CGCGGATCCTTACTTATCGTCGTCATCCTTGTAATCGAACTTGTAGTTCAGACCGAC |

**TABLE S2 | Primers for Q-PCR and luciferase reporter assay in this study.**

| **Gene Primer ID** (GenBank accession number ) | **Primer sequence (5’-3’)** | |
| --- | --- | --- |
| Porcine TNF-α (NM_214022) |  |  |
| Porcine TNF-α_qPCR_F | AGCCTCTTCTCCTTCCTCCTG |  |
| Porcine TNF-α_qPCR_R | GAGACGATGATCTGAGTCCTTGG |  |
| Porcine IL-12 p40 (NM_002187) |  |  |
| Porcine IL-12 p40_qPCR_F | GTGAGGTCTTAGGCTCTGGC |  |
| Porcine IL-12 p40_qPCR_R | AACCTCGCCTCCTTTGTGAC |  |
| Porcine IL-6 (NM_214055.1) |  |  |
| Porcine IL-6_qPCR_F | CAGTCCAGTCGCCTTCTCCCT |  |
| Porcine IL-6_qPCR_R | CTCCTGATTGAACCCAGATTG |  |
| Porcine IL-1β (NM_214399.1) |  |  |
| Porcine IL-1β_qPCR_F | AAGATAACACGCCCACCCTG |  |
| Porcine IL-1β_qPCR_R | GAGTTTCCCAGGAAGACGGG |  |
| Porcine β-actin_qPCR_F | GCGCGGCTACAGCTTCACCA |  |
| Porcine β-actin_qPCR_R | GGGCAGCGGAACCGCTCATT |  |
| Porcine TNF-α-Promotor_F | CGGGGTACCCTACCCCTACCTGAGCCCTGT |  |
| Porcine TNF-α-Promotor_R | CCCAAGCTTAGATGGCTGGTCTCTCTCCTG |  |
| Porcine-TNF-α-WT-3’UTR_F | CGACGCCCCAGAGTGAGGACACCAGGG |  |
| Porcine-TNF-α-WT-3’UTR_R | CGAGCTCTCGCCACTGACCAGTAGGG |  |
| Porcine--TNF-α-130a-MT-3’UTR_F | AACGTGAACGGACATCGGGTGC |  |
| Porcine-TNF-α-130a-MT-3’UTR_R | TTCACGTTATATAAATAAGAG |  |
| Porcine--TNF-α-181-MT-3’UTR_F | ACTTACATCTCCAATGGCAGAGTGGGTA |  |
| Porcine-TNF-α-181-MT-3’UTR_R | ATGTAAGTCTAGTAAATAAAT |  |
| Porcine--TNF-α-301a-MT-3’UTR_F | AACGTGAAGTAAGCCCGTCGCCCACGTT |  |
| Porcine-TNF-α-301a-MT-3’UTR_R | TTCACGTTATATAAATAAGAG |  |
| Murine TNF-α (NM_013693) |  |  |
| Murine TNF-α_qPCR_F | TCGCTGAGGGAGCTTCTGCT |  |
| Murine TNF-α_qPCR_R | CATTTCTTTTCCAAGCGATCTTT |  |
| Murine IL-12 p40 (NM_001303244.1) |  |  |
| Murine IL-12 p40_qPCR_F | ATTACTCCGGACGGTTCACG |  |
| Murine IL-12 p40_qPCR_R | ACGCCATTCCACATGTCACT |  |
| Murine IL-6 (NM_031168.2) |  |  |
| Murine IL-6_qPCR_F | CACTTCACAAGTCGGAGGCT |  |
| Murine IL-6_qPCR_R | CTGCAAGTGCATCATCGTTGT |  |
| Murine IL-1β (NM_008361.4) |  |  |
| Murine IL-1β_qPCR_F | AACCTTTGACCTGGGCTGTC |  |
| Murine IL-1β_qPCR_R | AAGGTCCACGGGAAAGACAC |  |
| Murine β-actin_qPCR_F | GCGCGGCTACAGCTTCACCA |  |
| Murine β-actin_qPCR_R | GGGCAGCGGAACCGCTCATT |  |
| Murine TNF-α-Promotor_F | CGGGGTACCCTTTGAGGATACGTGGCCAC |  |
| Murine TNF-α-Promotor_R | CCCAAGCTTGGTGTCTTTTTCAGAGGGGCTC |  |
| Murine-TNF-α-WT-3’UTR_F | CGACGCAGCAGAAGCTCCCTCAGCGAG |  |
| Murine-TNF-α-WT-3’UTR_R | CGAGCTCATTTCTTTTCCAAGCGATCT |  |
| Murine-TNF-α-130a-MT-3’UTR_F | AACGTGAACGGACATCGGGTGC |  |
| Murine-TNF-α-130a-MT-3’UTR_R | TTCACGTTTATATAAATAAGAG |  |
| Murine-TNF-α-181-MT-3’UTR_F | ACTTACATCTCCAATGGCAGAGTGGGTA |  |
| Murine-TNF-α-181-MT-3’UTR_R | ATGTAAGTATAGTAAATAAAT |  |
| Murine-TNF-α-301a-MT-3’UTR_F | AACGTGAAATAAGCCCGTCGCCCACGTT |  |
| Murine-TNF-α-301a-MT-3’UTR_R | TTCACGTTTATAAATAAGAG |  |
| Murine-TNF-α-351-MT-3’UTR_F | AGAGTCCCAAGCCGCCGTCGCACACCGT |  |
| Murine-TNF-α-351-MT-3’UTR_R | TGGGACTCTTAGCCGACAAA |  |

**TABLE S3 | Sequences of porcine and murine miRNAs primers used in this study**

| **Gene primer ID** (miRBase accession number) | **Primer sequence (5’-3’)** |
| --- | --- |
| miR-27a-3p (MIMAT0000537) |  |
| miR-27a-3p FW | CTGATCTTCACAGTGGCTAAG |
| miR-27a-3p RT primer | GTTGGCTCTGGTGCAGGGTCCGAGGTATTCGCACCAGAGCCAACGCAGAA |
| miR-27b-3p (MIMAT0000126) |  |
| miR-27b-3p FW | CTGATCTTCACAGTGGCTAAG |
| miR-27b-3p RT primer | GTTGGCTCTGGTGCAGGGTCCGAGGTATTCGCACCAGAGCCAACGCAGAA |
| miR-125b (MIMAT0000135) |  |
| miR-125b FW | TCGTCCCTGAGACCCTAAC |
| miR-125b RT primer | GTTGGCTCTGGTGCAGGGTCCGAGGTATTCGCACCAGAGCCAACTCACAA |
| miR-130a-3p (MIMAT0000141) |  |
| miR-130a-3p FW | TCCGCCAGTGCAATGTTAAAA |
| miR-130a-3p RT primer | GTTGGCTCTGGTGCAGGGTCCGAGGTATTCGCACCAGAGCCAACATGCCC |
| miR-146a (MIMAT0000158) |  |
| miR-146a FW | CGGCTGAGAACTGAATTCCA |
| miR-146a RT primer | GTTGGCTCTGGTGCAGGGTCCGAGGTATTCGCACCAGAGCCAACAACCCA |
| miR-181c (MIMAT0000210) |  |
| miR-181c FW | TCGTAACATTCAACCTGTCG |
| miR-181c RT primer | GTTGGCTCTGGTGCAGGGTCCGAGGTATTCGCACCAGAGCCAACACTCAC |
| RNU6B RT primer | GTTGGCTCTGGTGCAGGGTCCGAGGTATTCGCACCAGAGCCAACAAAAATAT |
| RNU6B FW | TTCCTCCGCAAGGATGACACGC |
| Reverse primer | GTGCAGGGTCCGAGGT |
| ssc-miR-124-3p (MIMAT0002156) |  |
| ssc-miR-124-3p FW | CTGATCTAAGGCACGCGGTG |
| ssc-miR-124-3p RT primer | GTTGGCTCTGGTGCAGGGTCCGAGGTATTCGCACCAGAGCCAACTGGCAT |
| ssc-miR-125a (MIMAT0013897) |  |
| ssc-miR-125a FW | TCGGTCCCTGAGACCCTTTA |
| ssc-miR-125a RT primer | GTTGGCTCTGGTGCAGGGTCCGAGGTATTCGCACCAGAGCCAACCACAGG |
| ssc-miR-140-3p (MIMAT0006786) |  |
| ssc-miR-140-3p FW | TCGGTCTACCACAGGGTAGAA |
| ssc-miR-140-3p RT primer | GTTGGCTCTGGTGCAGGGTCCGAGGTATTCGCACCAGAGCCAACGTCCGT |
| ssc-miR-142a-3p (MIMAT0020362) |  |
| ssc-miR-142a-3p FW | GCAAGATGTAGTGTTTCCTACTT |
| ssc-miR-142a-3p RT primer | GTTGGCTCTGGTGCAGGGTCCGAGGTATTCGCACCAGAGCCAACCCATAA |
| ssc-miR-146b (MIMAT0010190) |  |
| ssc-miR-146b FW | GGCGCTGAGAACTGAATTCCA |
| ssc-miR-146b RT primer | GTTGGCTCTGGTGCAGGGTCCGAGGTATTCGCACCAGAGCCAACAGCCTA |
| ssc-miR-155 (MIMAT0022959) |  |
| ssc-miR-155 FW | CGGCTTAATGCTAATTGTGA |
| ssc-miR-155 RT primer | GTTGGCTCTGGTGCAGGGTCCGAGGTATTCGCACCAGAGCCAACACCCCTA |
| ssc-miR-181a (MIMAT0010191) |  |
| ssc-miR-181a FW | TCGGTAACAATCAACGCTGTCG |
| ssc-miR-181a RT primer | GTTGGCTCTGGTGCAGGGTCCGAGGTATTCGCACCAGAGCCAACAACTCA |
| ssc-miR-181b (MIMAT0002126) |  |
| ssc-miR-181b FW | TCCGTAACATTCATTGCTGTCG |
| ssc-miR-181b RT primer | GTTGGCTCTGGTGCAGGGTCCGAGGTATTCGCACCAGAGCCAACAACCCA |
| ssc-miR-181d (MIMAT0013888) |  |
| ssc-miR-181d FW | CCGTAACATTCATTGTTGTC |
| ssc-miR-181d RT primer | GTTGGCTCTGGTGCAGGGTCCGAGGTATTCGCACCAGAGCCAACAACCCA |
| ssc-miR-301a-3p (MIMAT0002138) |  |
| ssc-miR-301a-3p FW | CGCCCAGTGCAATAGTATTG |
| ssc-miR-301a-3p RT primer | GTTGGCTCTGGTGCAGGGTCCGAGGTATTCGCACCAGAGCCAACGCTTTG |
| ssc-miR-351-5p (MIMAT0000608) |  |
| ssc-miR-351-5p FW | CTGTCCCTGAGGAGCCCTT |
| ssc-miR-351-5p RT primer | GTTGGCTCTGGTGCAGGGTCCGAGGTATTCGCACCAGAGCCAACCAGGCT |
| mmu-miR-124-3p (MIMAT0000134) |  |
| mmu-miR-124-3p FW | CTGATCTAAGGCACGCGGTG |
| mmu-miR-124-3p RT primer | GTTGGCTCTGGTGCAGGGTCCGAGGTATTCGCACCAGAGCCAACGGCATT |
| mmu-miR-125a (MIMAT0000135) |  |
| mmu-miR-125a FW | TCGGTCCCTGAGACCCTTTA |
| mmu-miR-125a RT primer | GTTGGCTCTGGTGCAGGGTCCGAGGTATTCGCACCAGAGCCAACCACAGG |
| mmu-miR-140-3p (MIMAT0000152) |  |
| mmu-miR-140-3p FW | TCGGTCTACCACAGGGTAGAA |
| mmu-miR-140-3p RT primer | GTTGGCTCTGGTGCAGGGTCCGAGGTATTCGCACCAGAGCCAACCCGTGG |
| mmu-miR-142a-3p (MIMAT0000155) |  |
| mmu-miR-142a-3p FW | GCAAGATGTAGTGTTTCCTACTT |
| mmu-miR-142a-3p RT primer | GTTGGCTCTGGTGCAGGGTCCGAGGTATTCGCACCAGAGCCAACTCCATA |
| mmu-miR-146b (MIMAT0003475) |  |
| mmu-miR-146b FW | GGCGCTGAGAACTGAATTCCA |
| mmu-miR-146b RT primer | GTTGGCTCTGGTGCAGGGTCCGAGGTATTCGCACCAGAGCCAACAGCCTA |
| mmu-miR-155 (MIMAT0000165) |  |
| mmu-miR-155 FW | CGGCTTAATGCTAATTGTGA |
| mmu-miR-155 RT primer | GTTGGCTCTGGTGCAGGGTCCGAGGTATTCGCACCAGAGCCAACACCCCTA |
| mmu-miR-181a (MIMAT0000210) |  |
| mmu-miR-181a FW | TCGGTAACAATCAACGCTGTCG |
| mmu-miR-181a RT primer | GTTGGCTCTGGTGCAGGGTCCGAGGTATTCGCACCAGAGCCAACAACTCA |
| mmu-miR-181b (MIMAT0000673) |  |
| mmu-miR-181b FW | TCCGTAACATTCATTGCTGTCG |
| mmu-miR-181b RT primer | GTTGGCTCTGGTGCAGGGTCCGAGGTATTCGCACCAGAGCCAACAACCCA |
| mmu-miR-181d (MIMAT0004324) |  |
| mmu-miR-181d FW | CCGTAACATTCATTGTTGTC |
| mmu-miR-181d RT primer | GTTGGCTCTGGTGCAGGGTCCGAGGTATTCGCACCAGAGCCAACAACCCA |
| mmu-miR-301a-3p (MIMAT0000379) |  |
| mmu-miR-301a-3p FW | CGCCCAGTGCAATAGTATTG |
| mmu-miR-301a-3p RT primer | GTTGGCTCTGGTGCAGGGTCCGAGGTATTCGCACCAGAGCCAACGCTTTG |
| mmu-miR-351-5p (MIMAT0000609) |  |
| mmu-miR-351-5p FW | CTGTCCCTGAGGAGCCCTT |
| mmu-miR-351-5p RT primer | GTTGGCTCTGGTGCAGGGTCCGAGGTATTCGCACCAGAGCCAACCAGGCT |

Notes: miRNA without "mmu" or "ssc" presents that porcine and murine share the same sequence.

**TABLE S4 | miRNA mimics and Anti-miRNA used in this study**

| **Primer ID**  mimics control | **Primer sequence (5’-3’)**  UUCUCCGAACGUGUCACGUTT |
| --- | --- |
| mir-130a-3p mimics | CAGUGCAAUGUUAAAAGGGCAU |
| mir-146a mimics | UGAGAACUGAAUUCCAUGGGUU |
| ssc-mir-181a mimics | AACAUUCAACGCUGUCGGUGAGUU |
| ssc-mir-181b mimics | AACAUUCAUUGCUGUCGGUGGGUU |
| ssc-mir-301a-3p mimics | CAGUCCAAUAGUAUUGUCAAAGC |
| ssc-mir-351-5p mimics | UCCCUGAGGAGCCCUUUGAGCCUGA |
| mmu-mir-181a mimics | AACAUUCAACGCUGUCGGUGAGU |
| mmu-mir-181b mimics | AACAUUCAUUGCUGUCGGUGGGU |
| mmu-mir-301a-3p mimics | CAGUGCAAUAGUAUUGUCAAAGC |
| mmu-mir-351-5p mimics | UCCCUGAGGAGCCCUUUGAGCCUG |
| Anti-control | UCUACUCUUUCUAGGAGGUUGUGA |
| Anti-mir-130a-3p | AUGCCCUUUUAACAUUGCACUG |
| Anti-mir-146a | AACCCAUGGAAUUCAGUUCUCA |
| Anti-ssc-mir-181a | AACUCACCGACAGCGUUGAAUGUU |
| Anti-ssc-mir-181b | AACCCACCGACAGCAAUGAAUGUU |
| Anti-ssc-mir-301a-3p | GCUUUGACAAUACUAUUGGACUG |
| Anti-ssc-mir-351-5p | UCAGGCUCAAAGGGCUCCUCAGGGA |
| Anti-mmu-mir-181a | ACUCACCGACAGCGUUGAAUGUU |
| Anti-mmu-mir-181b | ACCCACCGACAGCAAUGAAUGUU |
| Anti-mmu-mir-301a-3p | GCUUUGACAAUACUAUUGCACUG |
| Anti-mmu-mir-351-5p | CAGGCUCAAAGGGCUCCUCAGGGA |

Notes: miRNA without "mmu" or "ssc" presents that porcine and murine share the same miRNA mimics or Anti-miRNA.
